# Cholinergic activity reflects reward expectations and predicts behavioral responses

**DOI:** 10.1016/j.isci.2022.105814

**Published:** 2022-12-16

**Authors:** Panna Hegedüs, Katalin Sviatkó, Bálint Király, Sergio Martínez-Bellver, Balázs Hangya

**Affiliations:** 1 Lendület Laboratory of Systems Neuroscience, Institute of Experimental Medicine, H-1083 Budapest, Hungary; 2 János Szentágothai Doctoral School of Neurosciences, Semmelweis University, H-1085 Budapest, Hungary; 3 Department of Biological Physics, Eötvös Loránd University, H-1117 Budapest, Hungary; 4 Department of Anatomy and Human Embryology, Faculty of Medicine and Odontology, University of Valencia, 46010 Valencia, Spain

**Keywords:** Biological sciences, Neuroscience, Behavioral neuroscience

## Abstract

Basal forebrain cholinergic neurons (BFCNs) play an important role in associative learning, suggesting that BFCNs may participate in processing stimuli that predict future outcomes. However, the impact of outcome probabilities on BFCN activity remained elusive. Therefore, we performed bulk calcium imaging and recorded spiking of identified cholinergic neurons from the basal forebrain of mice performing a probabilistic Pavlovian cued outcome task. BFCNs responded more to sensory cues that were often paired with reward. Reward delivery also activated BFCNs, with surprising rewards eliciting a stronger response, whereas punishments evoked uniform positive-going responses. We propose that BFCNs differentially weigh predictions of positive and negative reinforcement, reflecting divergent relative salience of forecasting appetitive and aversive outcomes, partially explained by a simple reinforcement learning model of a valence-weighed unsigned prediction error. Finally, the extent of cue-driven cholinergic activation predicted subsequent decision speed, suggesting that the expectation-gated cholinergic firing is instructive to reward-seeking behaviors.

## Highlights

• Predictive strength of sensory cues was controlled in Pavlovian conditioning
• BFCNs showed large responses to reward-predicting stimuli and surprising rewards
• A reinforcement learning model suggested valence-weighed, unsigned BFCN responses
• Cholinergic responses predicted reaction times

## Graphical abstract

**Figure:**
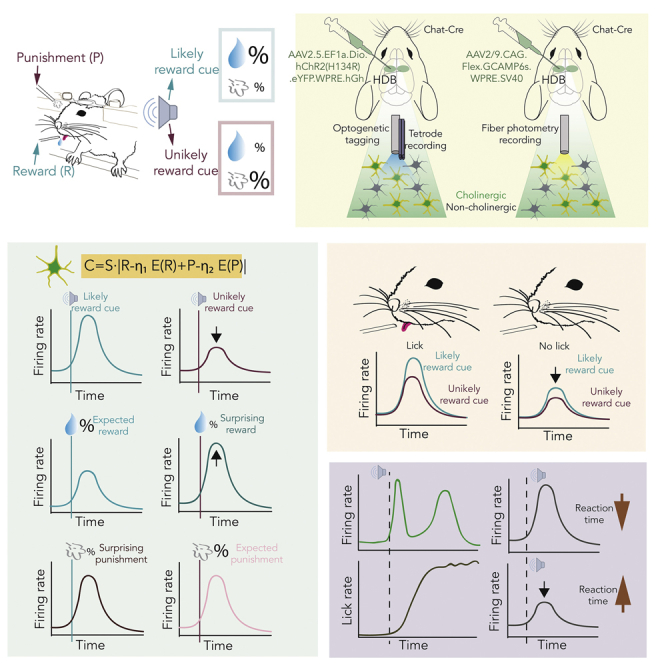

## Introduction

Cholinergic neurons of the basal forebrain are important for associative learning. This idea is supported by the selective cholinergic cell loss that parallels cognitive decline in patients with Alzheimer disease.^1^^,^^2^ Although lesion and pharmacology studies were confirmative,^3^^,^^4^^,^^5^ they cannot solve how BFCNs exert their control over learning. To address the mechanisms of the contribution of BFCNs to associative learning, it is important to investigate the behavioral correlates of BFCN activity at temporal resolutions comparable to the time scales of behaviorally relevant events animals and humans encounter.^6^^,^^7^ This has only become possible recently, enabled by the development of optogenetic and imaging tools.^8^^,^^9^^,^^10^^,^^11^

Selective cholinergic lesions of the basal forebrain were shown to impair learning in rodents^12^^,^^13^^,^^14^^,^^15^^,^^16^^,^^17^^,^^18^ and monkeys,^19^ and lesions of the basal forebrain in consequence of aneurysm rupture of the anterior cerebral or anterior communicating artery lead to severe learning impairments in humans.^20^ Previous studies of the basal forebrain have proposed that responses to behaviorally salient stimuli of cholinergic and/or noncholinergic basal forebrain neurons may underlie the involvement of the basal forebrain in learning.^9^^,^^10^^,^^11^^,^^21^^,^^22^ Specifically, cholinergic activation may lead to increased cortical acetylcholine release that induces plastic changes in sensory responses.^23^^,^^24^ A recent study connected the above pieces of evidence by bulk imaging of BFCNs during auditory fear learning.^11^ However, it is not yet known how BFCNs process sensory cues with different predictive features during learning, which could serve as a basis for differential behavioral responses to sensory events that forecast distinct outcomes. Therefore, a comprehensive model of cholinergic neuronal responses that subserve associative learning is also lacking. We set out to fill this knowledge gap by recording cholinergic activity in a probabilistic Pavlovian cued outcome task, which allowed us to directly control outcome probabilities and cue-outcome contingencies during learning.^25^ Of note, reward expectation can also be manipulated by reward size.^26^^,^^27^ However, since we hypothesized that BFCNs are sensitive to the outcome probabilities, we chose to manipulate reward probability instead, despite that this is harder to learn, as animals have to integrate over multiple trials to infer differences in probability, whereas reward size can be learned from a single trial.^28^

We imaged the bulk calcium responses of BFCNs using fiber photometry^29^ and recorded the activity of identified basal forebrain cholinergic neurons while mice were performing a head-fixed auditory probabilistic Pavlovian cued outcome task.^25^ BFCNs were activated by outcome-predicting stimuli, as well as delivery of reinforcement. Reward-predicting stimuli activated cholinergic neurons differentially in correlation with the likelihood of future reward, and subsequent reaction times were predicted by the level of this activation. BFCNs also showed stronger activation after unexpected compared with expected rewards. We show that these findings can be explained by a behavioral model of a stimulus-induced, valence-weighed prediction error, in which outcomes of opposite valence are differentially scaled by the animals. We did not observe robust firing rate changes of BFCNs following omissions of reinforcement, suggesting that the BFCN responses we observed were largely driven by sensory stimuli. Thus, these results suggest that the central cholinergic system broadcasts a stimulus-driven, valence-weighed prediction error signal that can instruct associative learning.

## Results

### Mice were trained on a probabilistic Pavlovian conditioning task

**Figure 1:**
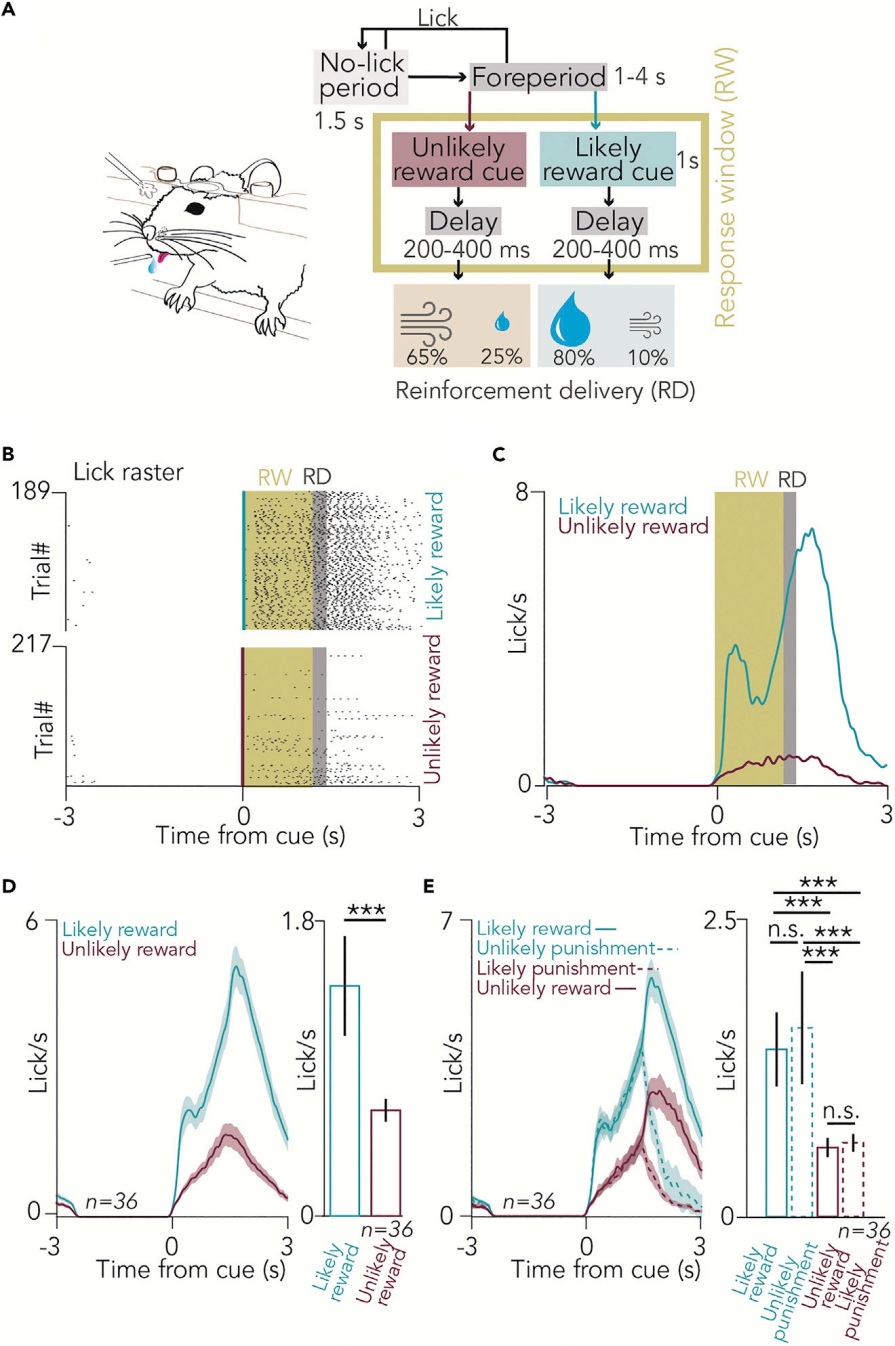
Mice were trained on a probabilistic Pavlovian conditioning task (A) Schematic illustration of the behavioral training and block diagram of the task. A variable foreperiod, in which the mouse was not allowed to lick, was followed by the presentation of one of two pure tones of well-separated pitch, which predicted reward, punishment, or nothing with different contingencies (“likely reward” and “unlikely reward” cues). (B) Raster plot of lick responses to the cues predicting likely reward (top) and unlikely reward (bottom) from an example session. Yellow shading, response window (RW); gray shading, reinforcement delivery (RD). (C) Peri-event time histograms (PETHs) of lick responses aligned to cue onset in the same session. (D) (Left) Average PETHs of lick responses of all sessions of all animals (n = 36 sessions). (Right) Statistical comparison of anticipatory lick rates in the RW in likely reward and unlikely reward trials (median ± SE of median, n = 36 sessions, p = 8.7697 × 10^−7^, Wilcoxon signed-rank test; ∗∗∗p < 0.001). (E) (Left) Average PETHs of lick responses of all sessions of all animals (n = 36 sessions), partitioned based on four possible outcomes: expected or surprising reward, expected or surprising punishment. (Right) Statistical comparison of anticipatory lick rates in the RW for the four possible outcomes (median ±SE of median, n = 36 sessions, from top to bottom, p = 1.4131 × 10^−6^, p = 2.6341 × 10^−6^, p = 0.8628, p = 6.8863 × 10^−7^, p = 1.2065 × 10^−6^, p = 0.9687, Wilcoxon signed-rank test; ∗∗∗p < 0.001; n.s., p > 0.05).

We trained mice (n = 11) on a head-fixed probabilistic Pavlovian cued outcome task (Figure 1A).^25^^,^^28^ During this associative learning task, two tones of different pitch (conditioned stimuli) predicted either water reward with 80% chance (10% punishment, 10% omission, “likely reward” cue) or a puff of air on the face with 65% probability (25% reward, 10% omission, “unlikely reward” cue; the contingencies reflect careful calibration to keep mice motivated for the task). Based on the cue that preceded behavioral feedback (unconditioned stimuli), both rewards and punishments could be either expected, or surprising. Mice learned this task, indicated by performing significantly more anticipatory licks after “likely reward” cues (Figures 1B–1E).

### BFCN population responses to conditioned and unconditioned stimuli

**Figure 2:**
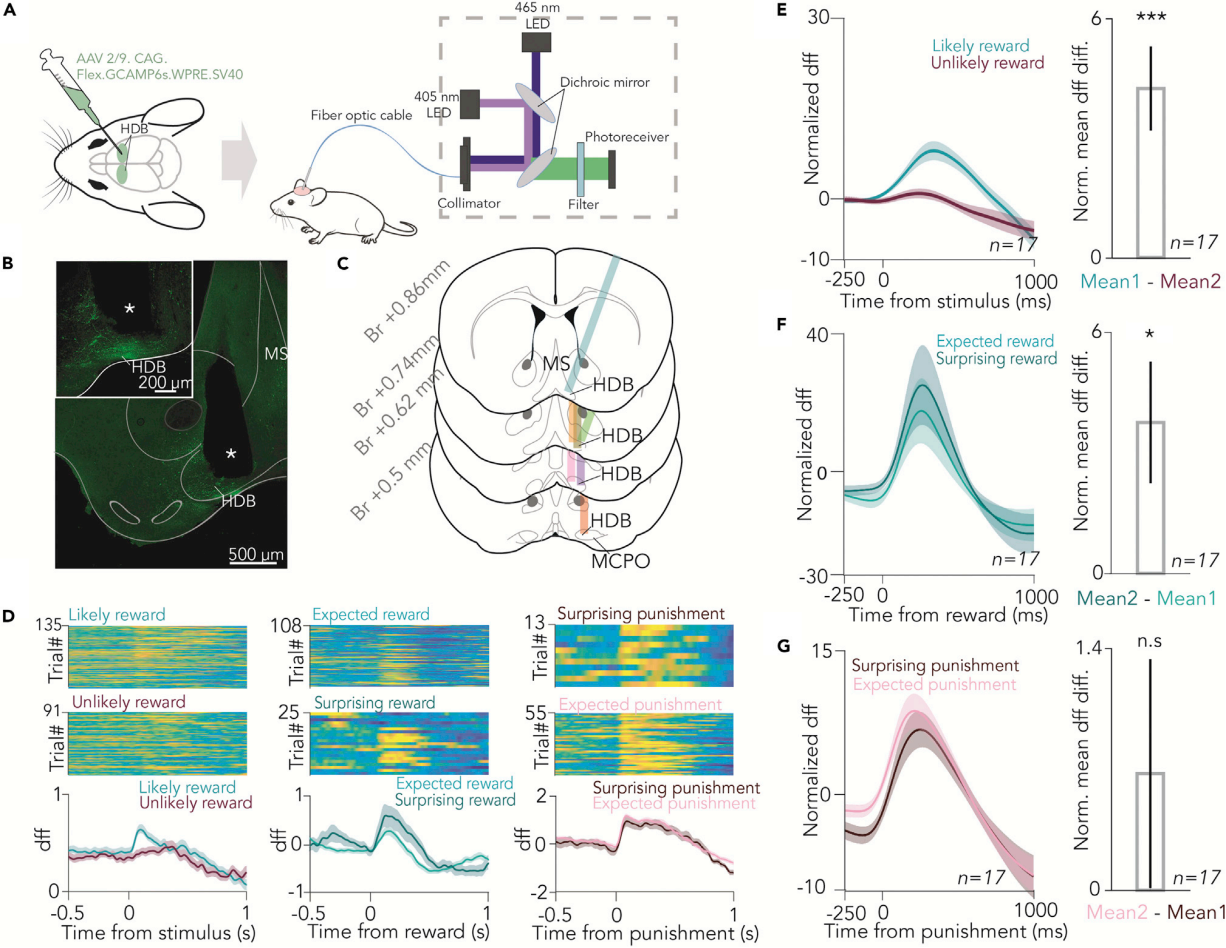
BFCN population responses to conditioned and unconditioned stimuli (A) Schematic diagram of bulk calcium imaging of HDB BFCNs in behaving mice. (B) Histological reconstruction of the optic fiber track in the HDB (white asterisk). Scale bar, 500 μm. Inset shows magnified view. Scale bar, 200 μm. (C) Optical fiber locations in all imaged mice (n = 7). Br, anteroposterior distance from Bregma. (D) Example session of bulk calcium imaging of cholinergic neurons from the HDB. (Left) dff signals were aligned to outcome-predicting conditioned stimuli. (Top) Trials with likely reward (unlikely punishment) cue; (middle) trials with unlikely reward (likely punishment) cue; (bottom) PETH. (Middle) dff signals were aligned to reward delivery. (Top) Trials with expected reward; (middle) trials with surprising reward; (bottom) PETH. (Right) dff signals were aligned to air puff punishments. (Top) Trials with surprising punishment. (Middle) Trials with expected punishment. (Bottom) PETH. (E) (Left) Average PETH of Z-scored dff aligned to outcome-predicting conditioned stimuli (n = 17 sessions). (Right) Bar graph of average normalized difference in dff after likely reward and unlikely reward cues. Median ±SE of median, ∗∗∗p < 0.001, p = 0.00029, Wilcoxon signed-rank test. (F) (Left) Average PETH of Z-scored dff aligned to expected and surprising reward (n = 17 sessions). (Right) Bar graph of average normalized difference in dff after surprising and expected reward. Median ±SE of median, ∗p < 0.05, p = 0.0129, Wilcoxon signed-rank test. (G) (Left) Average PETH of Z-scored dff aligned to expected and surprising punishment (n = 17 sessions). (Right) Bar graph of average normalized difference in dff after expected and surprising punishment. Median ±SE of median, n.s., p > 0.05, p = 0.0684, Wilcoxon signed-rank test. See also Figure S1.

We expressed GCaMP6s in BFCNs of the horizontal nucleus of the diagonal band of Broca (HDB) by injecting AAV2/9.CAG.Flex.GCAMP6s.WPRE.SV40 in ChAT-Cre mice (n = 7), expressing Cre recombinase driven by the choline acetyltransferase promoter selectively in cholinergic neurons,^10^^,^^30^^,^^31^ and implanted them with an optic fiber in the HDB. We performed bulk calcium imaging of HDB BFCNs while mice were performing the probabilistic Pavlovian task (Figures 2A–2C and S1). An excitation isosbestic wavelength of GCaMP was used to correct for non-calcium-dependent changes in fluorescence (e.g., bleaching and potential movement artifacts).^32^ We first asked whether BFCNs as a population responded to auditory cue stimuli that predicted outcomes with different contingencies. Fluorescent dff responses were aligned to cue presentations, revealing BFCN population calcium responses to outcome-predicting cue stimuli (Figures 2D and 2E). These responses were significantly larger for cues that predicted “likely reward” compared with cues predicting “likely punishment” (Figure 2D and 2E, p = 0.00029, Wilcoxon signed-rank test, n = 17 sessions). Based on published results of us and others,^8^^,^^9^^,^^10^^,^^11^ we expected BFCN calcium responses following the delivery of rewards and punishments as well. Indeed, when dff recordings were aligned to reinforcement, we observed robust cholinergic population responses to both water reward and air puff punishment (Figures 2F and 2G). Moreover, we found that BFCN responses to surprising rewards significantly exceeded those to expected rewards, although the observed difference was less than that of the cue responses (Figure 2F, p = 0.0129, Wilcoxon signed-rank test, n = 17 sessions). We did not find a significant difference between BFCN calcium responses to surprising vs. expected punishments (Figure 2G, p = 0.0684, Wilcoxon signed-rank test, n = 17 sessions).

### Optogenetic identification of basal forebrain cholinergic neurons during probabilistic Pavlovian conditioning

**Figure 3:**
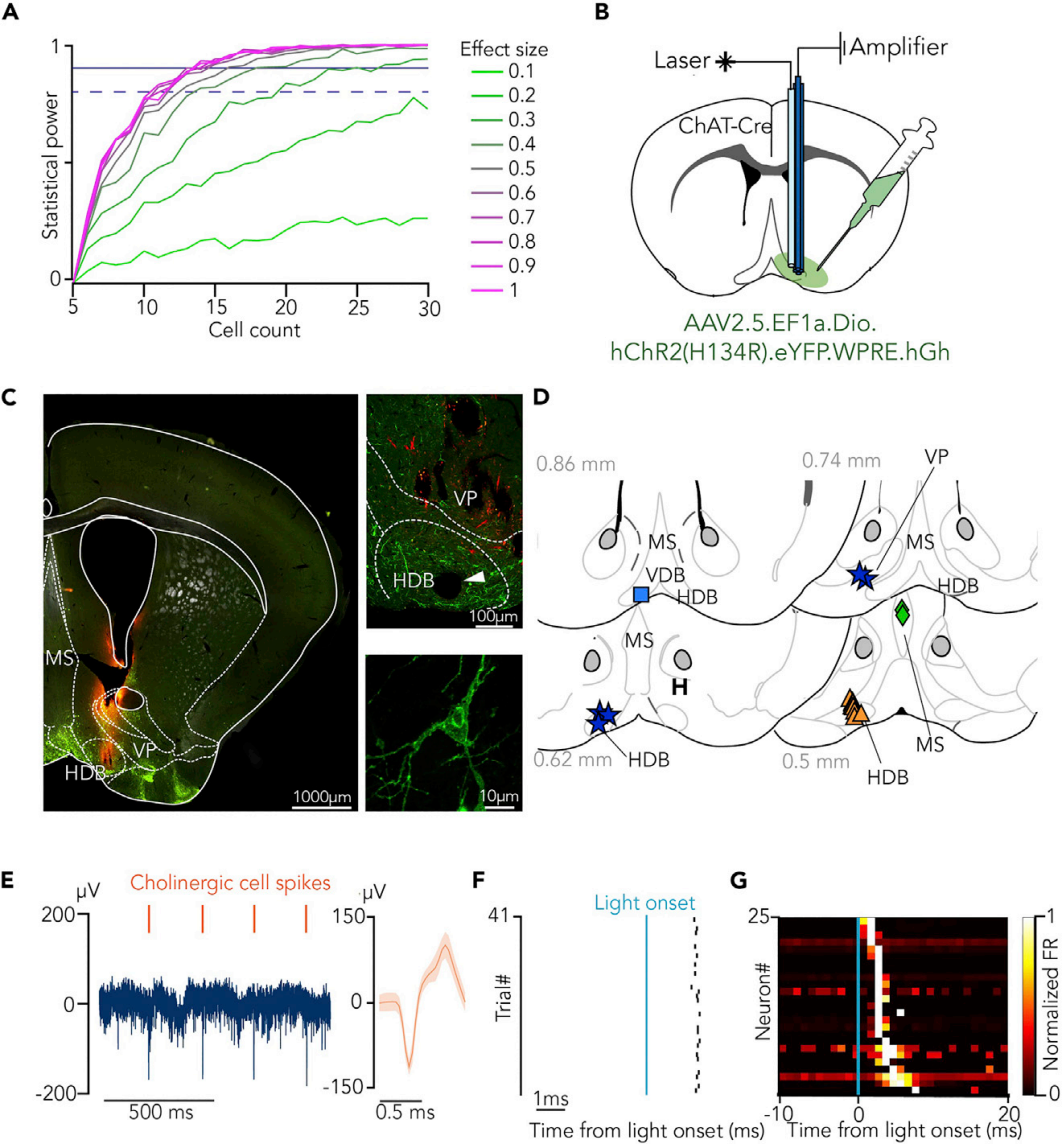
Optogenetic identification of basal forebrain cholinergic neurons during probabilistic Pavlovian conditioning (A) Statistical power as a function of cell count at different expected effect sizes. Dashed line, 80% power; solid line, 90% power. (B) Schematic drawing of optogenetic tagging. ChAT-Cre mice were injected with AAV2/5. EF1a.Dio.hChR2(H134R)-eYFP.WPRE.hGH. Eight moveable tetrodes were implanted in the HDB along with an optic fiber. (C) (Left) Coronal section from a ChAT-Cre mouse showing the distribution of cholinergic neurons (eYFP, green) and the tetrode track (DiI, red). Scale bar, 1000 μm. (Top right) Magnified view of the HDB. The white arrowhead points to the electrolytic lesion marking the tetrode tips. Scale bar, 100 μm. (Bottom right) Confocal image of a cholinergic neuron from the target area. Scale bar, 10 μm. (D) Reconstructed localization of all identified cholinergic neurons. Different markers correspond to individual mice. Numbers correspond to antero-posterior distance from Bregma, in mm. (E) (Left) Raw extracellular recording of an identified cholinergic neuron. (Right) Average waveform of the example cholinergic neuron. Orange marks above the recording indicate the cholinergic spikes. (F) Raster plot of an example cholinergic neuron showing short latency responses to 1-ms blue laser pulses. (G) Color-coded PETH of all identified cholinergic neurons aligned to laser pulse onset, sorted by response latency (black, no spikes; white, high firing rate). See also Figures S2 and S3.

Does the spiking of individual BFCNs show similar differential responses to conditioned and unconditioned stimuli according to different outcome expectations? We estimated that a sample of 14–20 identified BFCNs is sufficient to answer such a question with 80% statistical power (assuming a 30%–40% firing rate change corresponding to 0.3–0.4 predicted effect size, detectable in 60% of recorded neurons; the full procedure is available at https://github.com/hangyabalazs/statistical-power; Figure 3A). We expressed channelrhodopsin in BFCNs by injecting AAV.2.5.EF1a.DiO.hChR2(H134R).eYFP.WPRE.hGh in the basal forebrain of ChAT-Cre mice (n = 4) and implanted them with eight moveable tetrode electrodes and an optic fiber (Figures 3B and 3C), to optogenetically tag BFCNs in mice performing the probabilistic Pavlovian task.^10^^,^^11^ We recorded 25 optogenetically identified, ChAT-expressing BFCNs (p < 0.01, stimulus-associated spike latency test^33^) in task-performing mice (Figures 3D–3G, S2, and S3). Cholinergic neurons were recorded at a stage when the animals reached stable behavioral performance. Careful post-hoc histological reconstruction of the location of the recording electrodes showed that 21 of 25 neurons were recorded from HDB, whereas the remaining 4 of 25 neurons were in the medial septum (n = 2) and the ventral pallidum (n = 2; Figure 3D). Since these neurons exhibited similar responses to conditioned and unconditioned stimuli, they were treated as a single data set for this study; nevertheless, restricting data analyses to the HDB cholinergic neurons yielded similar results.

### Large cholinergic responses to reward-predicting cues, surprising rewards, and air puff punishments

**Figure 4:**
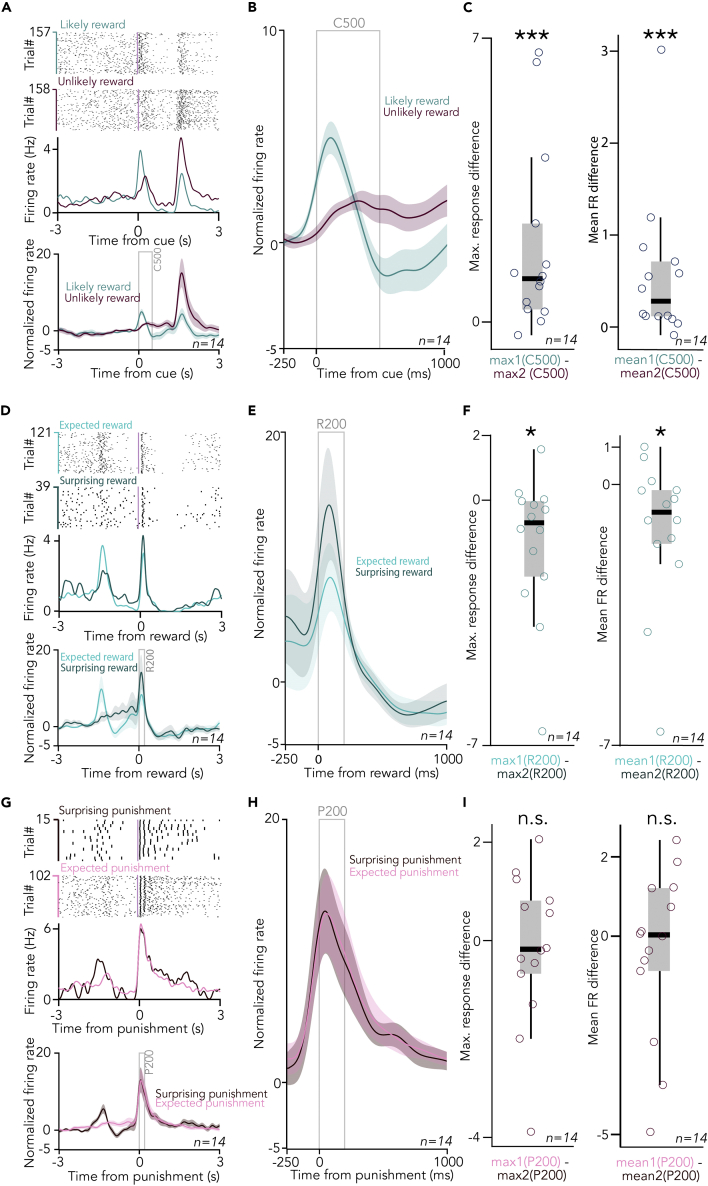
Cholinergic neurons respond more to reward-predicting cues and surprising reward (A) Top, raster plots (top) and PETHs (bottom) of an example BFCN aligned to cue onset, separately for the cues predicting likely reward/unlikely punishment (turquoise) vs. unlikely reward/likely punishment (purple). (Bottom) Average cue-aligned PETHs of identified BFCNs with >10 surprising reward trials (errorshade, mean ± SE; n = 14; see Figure S6 for all n = 25 neurons). (B) Average cue-aligned PETHs of identified BFCNs enlarged around cue presentations. (C) (Left) Difference in peak response after cues predicting likely reward and those predicting unlikely reward. ∗∗∗p < 0.001, p = 0.0008, Wilcoxon signed-rank test, n = 14. (Right) Difference in mean firing rate after cues predicting likely reward and those predicting unlikely reward. ∗∗∗p < 0.001, p = 0.00061, Wilcoxon signed-rank test, n = 14. Box-whisker plots show median, inter-quartile range, and non-outlier range. (D) (Top) Raster plots (top) and PETHs (bottom) of the same example BFCN as in (A), aligned to reward delivery, separately for rewards after the cue predicting likely reward (light turquoise, expected reward) vs. after the cue predicting unlikely reward (dark turquoise, surprising reward). (Bottom) Average reward-aligned PETHs of identified BFCNs with >10 surprising reward trials (errorshade, mean ± SE; n = 14; see Figure S6 for all n = 25 neurons). (E) Average reward-aligned PETHs of identified BFCNs enlarged around reward delivery times. (F) (Left) Difference in peak response after expected and surprising rewards. ∗p < 0.05, p = 0.0245, Wilcoxon signed-rank test, n = 14. (Right) Difference in mean firing rate after expected and surprising rewards. ∗p < 0.05, p = 0.02026, Wilcoxon signed-rank test, n = 14. Box-whisker plots show median, inter-quartile range, and non-outlier range. (G) (Top) Raster plots (top) and PETHs (bottom) of the same example BFCNs as in (A) and (D), aligned to punishment delivery, separately for punishments after the cue predicting likely reward (dark purple, surprising punishment) vs. after the cue predicting unlikely reward (light purple, expected punishment). (Right) Average punishment-aligned PETHs of identified BFCNs enlarged around punishment delivery times. (H) Average punishment-aligned PETHs of identified BFCNs with >10 surprising reward trials (errorshade, mean ± SE; n = 14; see Figure S6 for all n = 25 neurons). (I) (Left) Difference in peak response after expected and surprising punishments. n.s., p > 0.05, p = 0.7869, Wilcoxon signed-rank test, n = 14. (Right) Difference in mean firing rate after expected and surprising punishments. n.s., p > 0.05, p = 0.8393, Wilcoxon signed-rank test, n = 14. Box-whisker plots show median, inter-quartile range, and non-outlier range. See also Figures S4–S7.

We first asked whether individual BFCNs show spiking responses to auditory cue stimuli that predict outcomes with different probabilities. To address this, we aligned BFCN spikes to cue onset and examined raster plots and peri-event time histograms (PETHs) of individual BFCNs (see Figure S4 for a schematic representation of the analysis). We found that BFCNs responded to both auditory cues, with a median peak latency of 133.5 ms for the “likely reward” cue and 422 ms for the “unlikely reward” cue (Figures 4A, 4B, and S5A; interquartile range, 44.5–231 ms and 273–573.5 ms for the two cue types). To cover both peaks, we chose a 500-ms response window (C500), in which we compared BFCN responses to conditioned cue stimuli based on whether they signaled high or low probability of future reward. BFCNs showed 151% stronger responses to the “likely reward” cues based on a comparison of PETH peak responses in the C500 window (p = 0.0008, Wilcoxon signed-rank test; Figure 4C; including n = 14 neurons where mice encountered >10 surprising reward trials; see Figure S6 for all n = 25 neurons), which we also confirmed by spike-number-based statistics (p = 0.00061, Wilcoxon signed-rank test on BFCN firing rates in the C500 window; Figure 4C). Thus, BFCNs responded more to sensory stimuli that signaled high probability of reward.

Next, we tested whether individual BFCNs responded to the delivery of reward during Pavlovian conditioning, and whether this response depended on previous expectations about reward likelihoods conveyed by the two auditory cues. Therefore, we aligned the spike times of the same BFCNs to the time of reward delivery, again examining raster plots and PETHs (Figures 4D and 4E). We found that reward also elicited large BFCN responses, with a median peak latency of 86.5 and 82.7 ms for expected and surprising rewards, respectively (Figure S5B; interquartile range, 78.13–100.25 ms and 54.5–92.5 ms for expected and surprising rewards). To compare BFCN responses to expected vs. surprising rewards, we defined a 200-ms response window after reward delivery based on the above latency measurements (R200). We found that rewards that were less expected lead to significantly stronger cholinergic firing (69.3%, p = 0.0245, Wilcoxon signed-rank test on R200 response peaks; Figure 4F), also confirmed by firing rate comparison (p = 0.02026, Wilcoxon signed-rank test on BFCN firing rates in the R200 window; Figure 4F). These findings showed that BFCN responses were modulated by the expectation of reward.

We took a similar approach to investigate BFCN responses to the delivery of air puff punishments. BFCNs also responded with firing rate increase to punishment, with remarkably short peak latencies (Figures 4G, 4H, and S6C; median and interquartile range, 24.5 ms and 15.5–36 ms for surprising punishment and 24 ms and 15.5–32 ms for expected punishment), confirming previous results.^8^^,^^10^^,^^34^ When responses to surprising and expected punishments were directly compared in a 200-ms response window (P200), we did not find significant modulation by expectation (p = 0.7869, Wilcoxon signed-rank test on peak responses; Figure 4I; p = 0.8393, Wilcoxon signed-rank test on firing rates). We did not detect significant firing rate changes in either direction after omissions (Figure S7).

### Cholinergic responses are explained by a reinforcement learning model of stimulus-driven, valence-weighed, unsigned prediction error

The above-demonstrated differential BFCN responses to conditioned and unconditioned stimuli that reflected outcome expectations were suggestive of prediction error coding.^35^ Based on the positive-going BFCN responses following both reward and punishment, we assumed that BFCNs might represent an unsigned prediction error. If an outcome prediction error scaled positive and negative values equally, then it would track the expectation of reinforcement irrespective of valence. Thus, it would predict identical responses to conditioned cue stimuli that foreshadow reinforcement with a fixed probability, only sensitive to the rate of reinforcement omissions. However, cholinergic neurons showed stronger responses after cues predicting likely reward compared with those predicting unlikely reward but likely punishment. Therefore, our results suggest that BFCNs assign different weights to expected positive and negative outcomes, potentially related to the difference in absolute subjective values of the reinforcers. We did not observe BFCN responses to reinforcement omissions, suggesting that BFCN responses are driven by sensory stimuli, and thus a stimulus-driven, valence-weighed, unsigned prediction error model could explain BFCN spiking dynamics.

**Figure 5:**
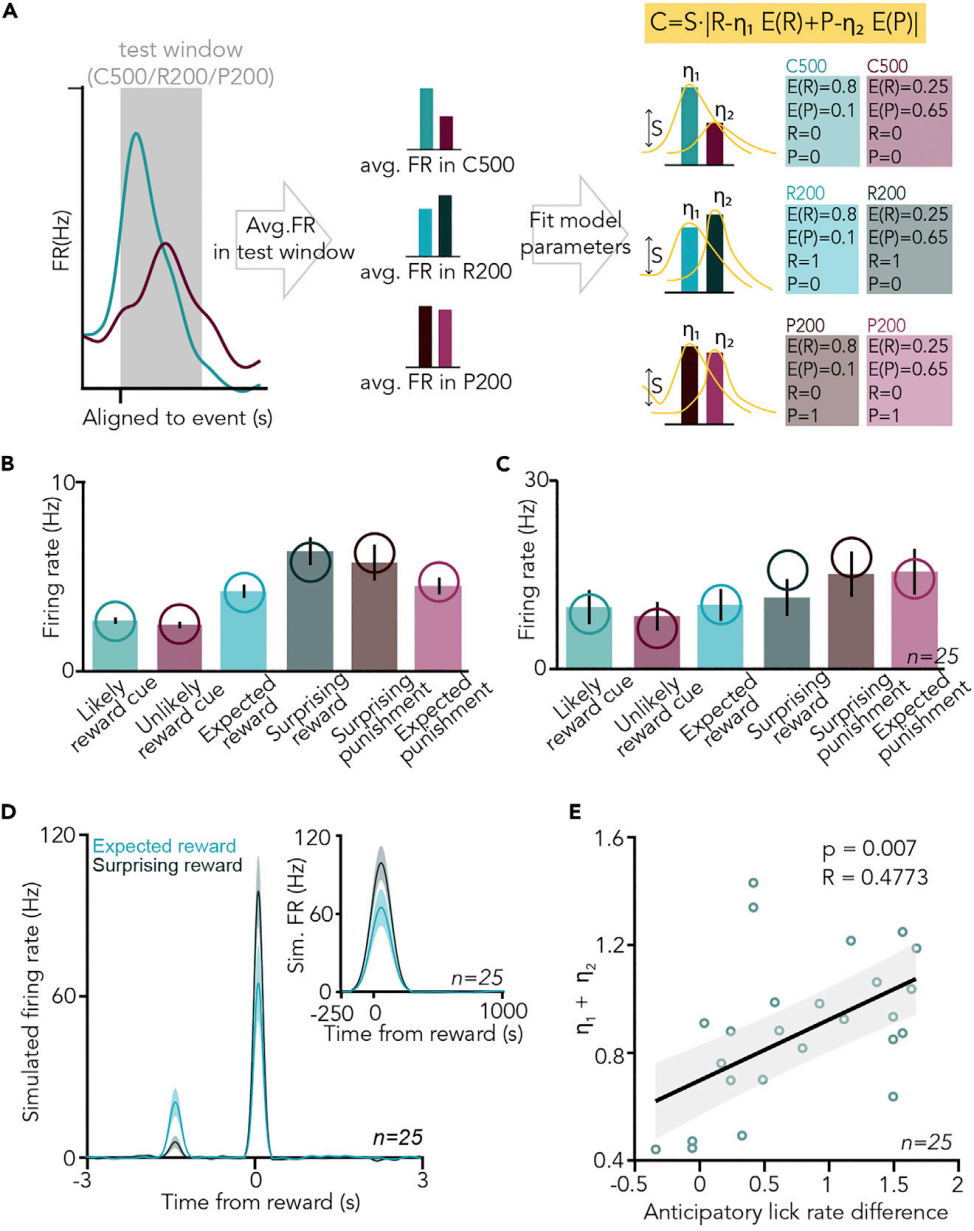
Cholinergic responses are explained by a reinforcement learning model of stimulus-driven, valence-weighed, unsigned prediction error (A) Schematic illustration of reinforcement learning model fitting on cholinergic neuronal data. Average firing rate (FR) values were fit by a three-parameter RL model incorporating task contingencies. (B) Firing rates of an example BFCN in 500-ms response windows after cue presentations and 200-ms response windows after reward or punishment, separated by trial type. Bar graphs represent mean ± SE over trials. Hypothetical firing rates corresponding to a best-fit RL model are overlaid, indicated by open circles. (C) Average firing rates of all identified BFCNs (n = 25) in the same response windows. Bar graphs represent mean ± SE over neurons. Average modeled firing rates are indicated by open circles. (D) Spike responses were simulated based on the best-fit RL models for each BFCN (see STAR Methods). PETHs were calculated the same way as for the real data and averaged over the modeled responses (n = 25). (E) The sum of the two model parameters that controls the differential sensitivity to reward and punishment expectations (*η*_*1*_*+ η*_*2*_) was correlated with the difference in anticipatory lick rate after likely reward vs. unlikely reward-predicting cues (R = 0.4773, Pearson’s correlation coefficient; p = 0.007, linear regression, F-test). See also Figure S8.

To test this, we implemented and fitted a simple three-parameter reinforcement learning (RL) model^35^^,^^36^ on cholinergic responses:

$$C=S\cdot \left|R-\eta_{1}E\left(R\right)+P-\eta_{2}E\left(P\right)\right|$$

where *C* represented cholinergic response, *S* was a scaling parameter accounting for different mean firing rates of BFCNs, *R* and *P* were actual, while *E(R)* and *E(P)* were expected reward and punishment determined by task contingencies. To take the assumed difference in the relative sensitivity to water reward and air-puff punishment into account, we introduced two weight parameters, *η*_*1*_ and *η*_*2*_ (0 ≤ *η*_*1*_, *η*_*2*_ ≤ 1), which could control how much BFCN responses were influenced by the expectation of positive and negative outcomes, respectively. Taking the absolute value of the sum of reward and punishment prediction error terms ensured positive-going cholinergic responses irrespective of valence, thus resulting in a simple model of unsigned reward prediction error. We found that this model fitted BFCN firing rate changes in response to the different cues and reinforcers defined by the C500, R200, and P200 response windows well (Figures 5A–5C), and significantly better than a control model in which the modeled expectations did not match the task contingencies (p = 0.0014 for all n = 25 BFCNs recorded; p = 0.0037 when only HDB cholinergic neurons were tested; Wilcoxon signed-rank test on the maximum likelihoods of the models; see STAR Methods).

We next simulated spike trains of individual BFCNs based on the best-fit RL models. Baseline firing was modeled by a Poisson process with a frequency matched to the baseline firing rate of the modeled BFCN, and simulated firing responses were added according to Gaussian distributions with a fixed delay after cue and reinforcement events, where the number of added spikes was determined by the best-fit RL model for each BFCN. When applying the same analyses on simulated spike trains as for the real data, we found that simulated PETHs qualitatively reproduced BFCN responses to cues and rewards (Figure 5D). These results further strengthen that the BFCN responses we observed are consistent with the representation of a stimulus-induced, valence-weighed, unsigned prediction error.

The best-fit *η*_*1*_ values were significantly larger than the best-fit *η*_*2*_ values, demonstrating stronger sensitivity of BFCN responses to reward than to punishment expectations (p = 0.0001, Wilcoxon signed-rank test; median ±SE of median, *η*_*1*_, 0.61 ± 0.04, *η*_*2*_, 0.37 ± 0.05). At the same time, the best-fit *η*_*2*_ values were significantly above 0.2, suggesting that mice learned to predict negative outcomes as well, reflected in their cholinergic responses according to the model (p = 0.0058, Wilcoxon signed-rank test). These parameters might reflect potential differences in the internal valuation of water reward and air puff punishment across animals and recording days, and also different sensitivity to reward expectation of individual BFCNs. We hypothesized that they reflect behavioral variability rather than heterogeneity across neurons, which would imply that these parameters show consistency within recording sessions and within individual mice. Indeed, we found smaller within- than across-mice differences in best-fit *η*_*1*_ parameters (p = 0.002, Mann-Whitney U test), and smaller within- than across-session differences in best-fit *η*_*2*_ parameters (p = 0.047, Mann-Whitney U test; n = 25; Figure S8). This suggests that best-fit scaling parameters for outcome expectations reflect inter-individual and/or behavioral differences, rather than differential sensitivity of individual BFCNs.

The perceived reward and punishment prediction errors are controlled by *η*_*1*_ and *η*_*2*_ in our model; therefore, they together determine the size of the unsigned outcome prediction error represented by BFCNs. If this can drive approach behaviors as previous studies suggested,^21^^,^^37^^,^^38^^,^^39^ then we would expect that the animals’ anticipatory licking behavior correlates with these model parameters. Indeed, we found that *η*_*1*_ as well as the sum of the two parameters (*η*_*1*_ *+ η*_*2*_), characterizing the cholinergic neurons’ sensitivity to momentary outcome prediction, correlates well with behavioral cue differentiation as indexed by anticipatory lick rate difference (p = 0.012, R = 0.52 and p = 0.056, R = 0.33 for *η*_*1*_ and *η*_*2*_, respectively; p = 0.007, R = 0.48 for *η*_*1*_ *+ η*_*2*_; n = 25; Figures 5E and S8; p = 0.0013 and R = 0.78 when calculated for the n = 14 neurons with >10 surprising reward trials; Pearson’s correlation coefficient, linear regression and one-sided F-test).

### Cholinergic responses predict reaction time

**Figure 6:**
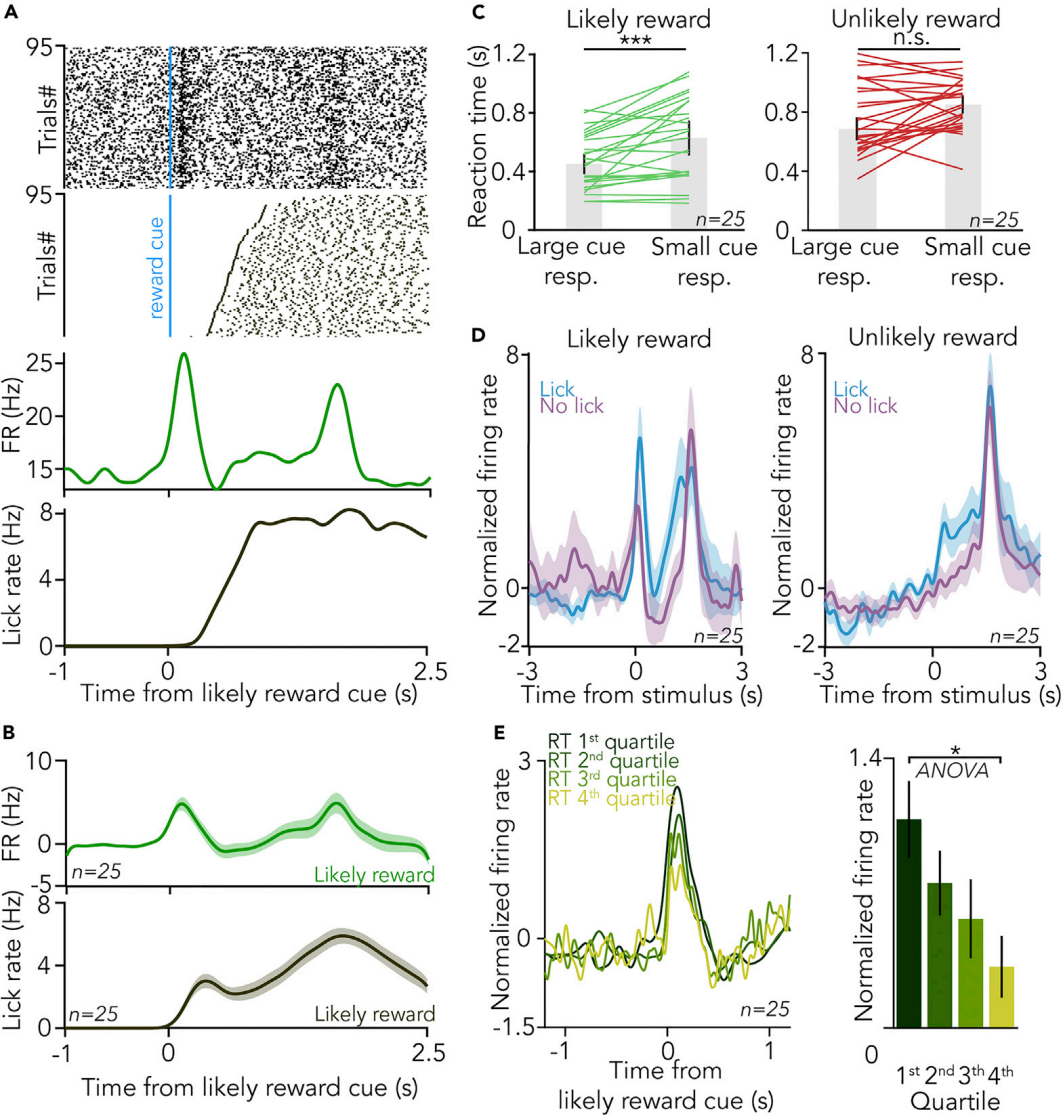
Cholinergic responses predict reaction time (A) (Top) Spike raster of cholinergic firing (top) and lick responses of the animal (bottom) aligned to the likely reward cues from an example session. (Bottom) Corresponding PETH of the cholinergic response (top) and licking activity (bottom). (B) Average PETH of cholinergic firing (top) and lick response (bottom) after the reward-predicting cue (n = 25 BFCNs). (C) Reaction time after large and small cue responses to the likely reward (left) and unlikely reward cue (right). Cue responses were divided by a median split. ∗∗∗p < 0.001, p = 0.00073 and n.s., p > 0.05, p = 0.05108, Wilcoxon signed-rank test, n = 25. Bar graphs represent median ±SE of median. (D) Average PETH of cholinergic responses to the likely reward and unlikely reward cue, separated based on the presence or absence of anticipatory lick response of the animal. (E) Stronger cholinergic response to the reward-predicting cue predicted faster reaction time. (Left) Average PETH of responses to the likely reward cue, partitioned to reaction time quartiles (1st quartile corresponds to shortest reaction time, in dark green). (Right) Bar graphs (mean ± SE) of the peak responses to the likely reward cue as a function of reaction time quartiles. ∗p < 0.05, p = 0.0314, one-way ANOVA. See also Figure S9.

The correlation of model parameters quantifying the animals’ sensitivity to outcome expectations with behavioral performance prompted us to further assess whether BFCN responses could predict animal behavior. BFCN responses to outcome-predicting cues consistently preceded the animals’ first licks (Figures 6A and 6B). When we aligned cholinergic spikes to the last lick before the foreperiod during which mice were not allowed to lick, cholinergic activity peaked before licking with a similar time course as during cue-related licking activity (Figure S9). These findings excluded that a potential “lick-driven” cholinergic activity could confound the results and instead indicated that cholinergic activity had the potential to influence behavioral responses of mice performing the task. Indeed, we found that larger cholinergic cue responses were followed by faster reactions (p = 0.00073 and p = 0.05108 for “likely reward” and “unlikely reward” cues, respectively; Wilcoxon signed-rank test; Figure 6C). In accordance, cholinergic cue responses were larger when mice were licking after the cue (p = 0.048 and p = 0.023 for “likely reward” and “unlikely reward” cues, respectively; Wilcoxon signed-rank test; Figure 6D). Since lick responses can be taken as an indication of mice expecting reward, these results are consistent with cholinergic reward expectation coding. Next, we divided the trials into four quartiles according to mice’s reaction times after cue onset. In line with the above results, we found that faster lick responses were preceded by stronger cholinergic firing (Figure 6E, p = 0.0314, one-way ANOVA). This was also reflected in a significant negative trial-by-trial correlation of BFCNs’ firing rate after the reward-predicting cue and animal reaction time (R = −0.45, p = 0.034; Pearson’s correlation coefficient, linear regression and one-sided F-test). In sum, these results indicate that cue responses of BFCNs predict reaction times, suggesting that cholinergic outcome prediction coding affects behavioral responses.

## Discussion

Cholinergic neurons of the basal forebrain respond to behaviorally salient events.^8^^,^^9^^,^^10^^,^^11^^,^^22^^,^^34^^,^^40^^,^^41^ To better understand the nature of these responses, we investigated whether activity patterns elicited by outcome-predictive stimuli and behavioral feedback are consistent with a prediction error hypothesis. By investigating the responses of BFCN populations using bulk calcium imaging and of individual BFCNs by optogenetic tagging in a probabilistic Pavlovian cued outcome task, we found that BFCNs showed strong activation after reward-predicting stimuli, and larger responses to surprising than to expected rewards. These results were consistent with a simple RL model of a stimulus-driven, valence-weighed, unsigned reward prediction error. The model also demonstrated that while BFCNs responded with firing rate increase to events of both positive and negative valence, they also reflected different behavioral sensitivity to positive and negative expectations. Finally, BFCN responses were found to likely influence behavioral performance, as mice showed faster responses after stronger cholinergic activation.

Temporal difference reinforcement learning (TDRL) models were successful in explaining the reward prediction errors represented by the dopaminergic system.^35^^,^^36^^,^^42^ The presence of a reward response modulated by expectation and responsiveness to reward-predicting sensory stimuli suggested that cholinergic signals may also be related to prediction errors; however, consistently positive-going responses for punishment indicated that this prediction error signal may be unsigned. A model that puts the same positive weight on both aversive and appetitive outcomes tracks the expectation of a reinforcement irrespective of its valence; therefore, it would predict identical responses for reward- and punishment-predicting cues if omission rate is constant. However, BFCNs clearly preferred reward-predicting stimuli, suggesting differential representation of rewards and punishments. Therefore, we implemented an RL model and fitted parameters capturing differential weighing based on valence. We found that this model reliably predicted average BFCN responses to cues, rewards, and punishments. It also reproduced larger BFCN responses to cues that foreshadowed rewards with high probability, as well as to surprising, when compared with expected rewards. The best-fit model indicated non-zero weights for both reward and punishment expectation, suggesting the behavioral anticipation of both types of outcomes, but with a significantly larger weight on the expectation of reward, suggesting that the outcome prediction error cholinergic neurons represented was indeed unequally weighed.

What may be the function of this fast prediction error signal? The cholinergic system has long been known to strongly influence cortical plasticity.^3^^,^^43^^,^^44^^,^^45^^,^^46^^,^^47^ A line of studies has demonstrated that pairing auditory stimuli with cholinergic stimulation reorganizes cortical sensory representations, known by the term “receptive field plasticity.”^23^^,^^24^ Furthermore, recent studies showed that cholinergic inputs may even endow primary sensory cortices with non-sensory representations not expected previously.^48^^,^^49^ In particular, Liu et al. showed that optogenetic activation of cholinergic fibers in the visual cortex entrained neural responses that mimicked behaviorally conditioned reward timing activity.^50^ It was also demonstrated that the cholinergic system exerts a rapid, fine-balanced control over plasticity at millisecond timescales, stressing the importance of timing even for neuromodulatory systems.^51^^,^^52^^,^^53^ This effect on plasticity might have a fundamental impact on associative learning at the behavioral level,^54^ also suggested by recent advances in the fear learning field.^11^^,^^22^^,^^55^^,^^56^

Indeed, we found that the best-fit model parameters were correlated with the difference in the animals’ anticipatory lick rate indicating learning performance. Moreover, cholinergic responses to reward-predicting cues predicted behavioral responses and reaction time, fitting in a more general scheme of basal forebrain control over response speed to motivationally salient stimuli.^21^^,^^37^^,^^45^ Therefore, we propose that a rapid acetylcholine-mediated cortical activation, scaled by unsigned outcome prediction error, tunes synaptic plasticity in the service of behavioral learning. This idea is supported by strong theories that associated prediction errors and cholinergic activity with learning and memory.^57^^,^^58^^,^^59^ Nevertheless, the functions of cholinergic effects probably go beyond learning, and BFCNs may control many aspects of behavior including arousal or alertness,^21^^,^^31^^,^^40^^,^^45^^,^^60^^,^^61^^,^^62^^,^^63^^,^^64^ attention,^3^^,^^54^^,^^65^^,^^66^^,^^67^ and vigilance.^68^^,^^69^^,^^70^

The activity of cholinergic neurons shares strong similarities with dopaminergic neurons in response to reward and reward-predicting cues.^35^^,^^71^^,^^72^^,^^73^ Reward-predicting cues evoke an increase in firing rate, which is stronger for more likely rewards. Reward itself also elicits cholinergic firing, but less so if the reward is more expected. However, cholinergic neurons differ from dopaminergic neurons in their response to punishment. Dopaminergic neurons can respond to aversive stimuli with either increased or decreased firing,^74^^,^^75^^,^^76^ whereas cholinergic neurons consistently respond with a fast, precisely timed response to air puffs. Therefore, the positive-going response of BFCNs irrespective of valence, sensitive to outcome probabilities for cues and rewards, suggests that compared with the reward prediction error signal dopaminergic neurons encode, BFCNs represent an unsigned outcome prediction error. Importantly, bursting basal forebrain neurons with similar coding properties have been uncovered in primates,^39^ suggesting that at least part of those neurons might be cholinergic.

Cholinergic neurons appeared to respond faster than dopaminergic neurons; however, response timing may depend on seemingly subtle details of the behavioral paradigm. Altogether, BFCNs appear to provide a faster but less specific response to salient stimuli, which is likely broadcasted to large cortical areas innervated by cholinergic fibers.^77^^,^^78^ In contrast, calculations related to value that are represented in the dopaminergic system may require more processing time and result in somewhat delayed, albeit more specific representations. Nevertheless, direct comparisons of cholinergic and dopaminergic neurons in the same experiment will be necessary to reveal the differential functions of these major neuromodulatory systems.

### Limitations of the study

A valence-weighed unsigned prediction error hypothesis predicts stronger response to unexpected than to expected punishment; the larger the weight of the punishment, the stronger the difference. We did not find a significant difference, which could be due to the lower weight of punishment that decreased statistical power, or a theoretical deviation from a full-fledged outcome prediction error.

Additionally, an unsigned prediction error signal predicts a firing rate increase after omitted reward. Note that unsigned prediction error variables only take non-negative values, and thus all unexpected changes in state value result in increased values due to the absolute value operator; however, different models that predict a firing rate decrease after omission are also conceivable. We tested an alternative model that included omission-related activity (see STAR Methods), but we found that this model was statistically indistinguishable from our original model based on our data set, suggesting that larger amounts of data are required to resolve this question. Also, given the phasic nature of cholinergic reinforcement responses comprising often very few (sometimes only one) but precisely timed action potentials,^10^ it is expected that an omission response, where there is no sensory stimulus to align to, is very hard to detect in single neurons. Indeed, a recent study demonstrated positive-going omission responses in HDB cholinergic neurons using fiber photometry.^41^ Alternatively, the cholinergic system may be sensitive to external sensory stimuli but not to absence of an expected stimulus, in line with its strong bottom-up anatomical inputs conveying sensory signals,^79^ likely gated via local inhibitory neurons that may relay expectation information.^80^ A recent study demonstrated topographic variations in cholinergic responses to salient events,^41^ which could also contribute to these ambiguities.

## STAR★Methods

### Key resources table

**Table:** 

REAGENT or RESOURCE	SOURCE	IDENTIFIER
**Bacterial and virus strains**		

AAV2/5.EF1a.DiO.hChR2(H134R).eYFP.WPRE.hGh	https://www.addgene.org/	20298-AAV5
AAV2/9.CAG.Flex.GCAMP6s.WPRE.SV40	https://www.addgene.org/	100842-AAV9

**Chemicals, peptides, and recombinant proteins**		

Isoflurane	https://vetcentre.com/	N/A
Ketamine	https://vetcentre.com/	N/A
Xylazine	https://vetcentre.com/	N/A
Buprenorphine	http://www.richter-pharma.com/	N/A
Neomycin	Local pharmacy	N/A
Lidocaine	Local pharmacy	N/A
Betadine	Local pharmacy	N/A
Promethazine	https://vetcentre.com/	N/A
Paraformaldehyde	https://taab.co.uk/	Cat#P001
Fluorescent dye (1,1'-Dioctadecyl-3,3,3',3'-Tetramethylindocarbocyanine Perchlorate, DiI)	https://www.thermofisher.com	Cat#D282
Aqua-Poly/Mount mounting medium	https://www.polysciences.com	Cat#18606
Metabond dental cement	http://www.parkell.com/	Cat#S380
Jet Set-4 Denture Repair Powder and Liquid	https://www.langdental.com/	N/A
Eye ointment	https://www.laboratoires-thea.com	N/A

**Deposited data**		

Electrophysiology and fiber photometry	https://doi.org/10.5061/dryad.p5hqbzkrv	https://doi.org/10.5061/dryad.p5hqbzkrv

**Experimental models: Organisms/strains**		

ChAT-Cre Bl6 mice	https://www.jax.org/	#006410

**Software and algorithms**		

Matlab 2016a	https://mathworks.com/	N/A
Algorithms for behavioral analysis	https://github.com/hangyabalazs/cholinergic_Pavlovian_analysis	N/A
Algorithms for closed loop behavioral control	https://github.com/hangyabalazs/Bpod_r0_5	CuedOutcomeTask
Code for simple data analysis of lick responses and neural recordings	https://github.com/hangyabalazs/CellBase	N/A
OPETH	https://github.com/hangyabalazs/opeth	SCR_018022
MClust 3.5	https://redishlab.umn.edu/mclust	v. 3.5.

**Other**		

Surgical forceps	https://www.finescience.com/en-US/	11151–10
Standard surgical scissors	https://www.finescience.com/en-US/	14060–10
Scraper	https://www.finescience.com/en-US/	10075–16
Headbar	Custom made	N/A
Headbar holders	Custom made	N/A
Lick port	https://www.shapeways.com/	N/A
Infrared sensor and emitter	https://www.digikey.com/	480-1958-ND and 480-1969-ND
Speaker	https://www.digikey.com/	668-1447-ND
Camera	https://www.flir.eu/iis/machine-vision/	FL3-U3-32S2M-CS
Bpod	https://sanworks.io/	1027
Teensy 3.2	https://www.pjrc.com/teensy/	N/A
Plastic tubing	https://www.thermofisher.com/	Cat#8001–0102 and Cat#8001–0204
Polyimide tubing	https://www.warneronline.com/	Cat#64–0755/PE-160
Feeding needle	https://www.finescience.com/en-US/	18060–20
Bulldog serrefine	https://www.finescience.com/en-US/	18050–28
Bone rongeour	https://www.finescience.com/en-US/	16012–12
Fiber Photometry System	https://neuro.doriclenses.com/	N/A
Data Acquisition System	https://openephys.org/acquisition-system/	Version#2.4
RHD 32-channel headstage	https://intantech.com	Part#C3314
RHD standard SPI interface cable	https://intantech.com	Part#3206
Ø400 μm Core, 0.50 NA FC/PC to Ø1.25 mm Ferrule Patch Cable, 1 m Long	https://www.thorlabs.de/	M127L01
Vibratome	https://www.leicabiosystems.com	2100S
Confocal microscope	https://www.microscope.healthcare.nikon.com/	C2
Stimulus isolator	https://www.superte.ch/	IBP-7c

### Resource availability

#### Lead contact

Further information and requests for resources should be directed to and will be fulfilled by the lead contact, Balázs Hangya (hangya.balazs@koki.hu).

#### Materials availability

This study did not generate new unique reagents.

### Experimental model and subject details

Adult (over two months old) male ChAT-Cre mice (The Jackson Laboratory, RRID: IMSR_JAX:006410) were used (n = 11). All experiments were approved by the Animal Care and Use Committee of the Institute of Experimental Medicine and the Committee for Scientific Ethics of Animal Research of the National Food Chain Safety Office (PE/EA/675–4/2016; PE/EA/1212–5/2017; PE/EA/864–7/2019) and were performed according to the guidelines of the institutional ethical code and the Hungarian Act of Animal Care and Experimentation (1998; XXVIII, section 243/1998, renewed in 40/2013) in accordance with the European Directive 86/609/CEE and modified according to the Directive 2010/63/EU.

### Method details

#### Tetrode implantation surgery

Mice were implanted using standard stereotaxic surgery techniques with miniaturized microdrives housing 8 tetrodes and an optic fiber.^10^^,^^33^^,^^81^ Briefly, mice were anesthetized by a mixture of ketamine and xylazine (83 and 17 mg/kg, respectively, dissolved in 0.9% saline). The skin was shaved and disinfected with Betadine, subcutaneous tissues were infused with Lidocaine, eyes were protected with eye ointment (Laboratories Thea) and mice were placed in a stereotaxic frame (Kopf Instruments). The skull was cleaned, and a craniotomy was drilled above the horizontal diagonal band of Broca (HDB, antero-posterior 0.75 mm, lateral 0.60 mm; n = 4) or the medial septum (MS, antero-posterior 0.90 mm, lateral 0.90 mm, 10 degrees lateral angle; n = 1). Virus injection (AAV2/5.EF1a.Dio.hChR2(H134R)-eYFP.WPRE.hGH; HDB, dorso-ventral 5.00 and 4.70 mm, 300 nL at each depth; MS, dorsoventral 3.95, 4.45 and 5.25 mm, 200 nL at each depth) and drive implantation was performed according to standard techniques.^10^^,^^33^ Ground and reference electrodes were implanted to the bilateral parietal cortex. Mice received analgesics (Buprenorphine, 0.1 mg/kg), local antibiotics (Neomycin) and were allowed 10 days of recovery before starting behavioral training.

#### Optical fiber implantation surgery

Mice (n = 7) were implanted using standard stereotactic surgery techniques described in the previous section. Following virus injection (AAV2/9.CAG.Flex.GCAMP6s.WPRE.SV40; HDB, 300 nL each side, antero-posterior 0.75 mm, lateral 0.60 mm; dorso-ventral 5.00 and 4.7 mm), 400 μm core diameter optic fibers with ceramic ferrules were implanted bilaterally (HDB, antero-posterior 0.75 mm, lateral 0.60 mm; 0 and 20 degrees lateral angle on the two sides). Optical fiber implantation was performed similarly to tetrode drive implantation. Mice received analgesics (Buprenorphine, 0.1 mg/kg), local antibiotics (Neomycin) and were allowed 10 days of recovery before starting behavioral training.

#### Behavioral training

Mice were trained on a head-fixed probabilistic auditory Pavlovian conditioning task^28^ in a custom-built behavioral setup that allowed millisecond precision of stimulus and reinforcement delivery (described in^7^). Mice were water restricted before training and worked for small amounts of water reward (5 μL) during conditioning. Pure tones of one second duration predicted likely reward/unlikely punishment or unlikely reward/likely punishment based on their pitch (12 kHz tone predicted 80% water reward, 10% air-puff punishment, 10% omission; 4 kHz tone predicted 25% water reward, 65% air-puff punishment, 10% omission in n = 6 mice; opposite cue contingencies were used in n = 5 mice, see Figures S1 and S3; 50–50% of the two cue tones were mixed randomly; all cue tone intensities were set at 50 dB sound pressure level). The animal was free to lick a waterspout after tone onset and individual licks were detected by the animal’s tongue breaking an infrared photobeam. After an additional 200–400 ms post-stimulus delay, the animal received water reward, air-puff punishment or omission, pseudorandomized according to the above contingencies. The next trial started after the animal stopped licking for at least 1.5 s. The stimulus was preceded by a 1–4 s foreperiod according to a truncated exponential distribution, in order to prevent temporal expectation of stimulus delivery. If the mouse licked in the foreperiod, the trial was restarted. We used the open source Bpod behavioral control system (Sanworks LLC, US) for operating the task. Behavioral performance of the task did not depend on the identity (frequencies) of the conditioned stimuli (Figure S1).

The aversive quality of air-puffs depends on the exact experimental settings. We applied 200 ms long puffs at 15 psi pressure (within the range of parameters used for eyeblink conditioning^82^). We demonstrated that mice consistently choose water without air-puff over water combined with air-puff, showing that air-puffs are aversive under these circumstances (see Figures 2C and 2D in ^10^). We also demonstrated that water and air-puff are accompanied by different auditory signals in our setup, thus making sensory response generalization unlikely to explain BFCN responses (see Figure S2A in ^28^).

#### Fiber photometry imaging

Bilateral fluorescent calcium imaging was performed using a dual fiber photometry setup (Doric Neuroscience) and visualized during training sessions using Doric Studio Software. Two LED light sources (465 nm, 405 nm) were channeled in fluorescent Mini Cubes (iFMC4, Doric Neuroscience). Light was amplitude-modulated by the command voltage of the two-channel LED driver (LEDD_2, Doric Neuroscience, the 465 nm wavelength light was modulated at 208 Hz and 405 nm wavelength was modulated at 572 Hz). Light was channeled into 400 μm diameter patch cord fibers and was connected to optical fiber implants during training sessions. The same optical fibers were used to collect the bilateral emitted fluorescence signal, which were detected with 500–550 nm fluorescent detectors integrated in the Mini Cubes. Emitted signals were sampled at 12 kHz, decoded in silico and saved in a ∗.csv format.

#### Chronic extracellular recording

We used the open ephys data acquisition system^83^ for spike data collection. A 32-channels Intan headstage (RHD2132) was connected to the Omnetics connector on the custom-built microdrive. Data was transferred through digital SPI cables (Intan) to the Open Ephys board and saved by the Open Ephys software, digitized at 30 kHz.

#### Optogenetic tagging

The custom microdrives were equipped with a 50 μm core optic fiber (Thorlabs) that ended in an FC connector (Precision Fiber Products). This was connected with an FC-APC patch chord during recording. For optogenetic tagging, 1 ms laser pulses were delivered (473 nm, Sanctity) at 20 Hz for 2 seconds, followed by 3 seconds pause, repeated 20–30 times. Light-evoked spikes and potential artifacts were monitored online using the OPETH plugin (SCR_018022)^84^ and laser power was adjusted as necessary to avoid light-induced photoelectric artifacts and population spikes that could mask individual action potentials. Significance of photoactivation was assessed during offline analyses by the SALT test based spike latency distributions after light pulses, compared to a surrogate distribution using Jensen-Shannon divergence (information radius).^33^^,^^85^ Neurons with p < 0.01 were considered light-activated, and thus cholinergic. Cholinergic neurons recorded on the same tetrode within 200 μm dorso-ventral distance were compared by waveform correlation and autocorrelogram similarity,^28^^,^^86^ and similar units were counted towards the sample size only once.

#### Histology

After the last behavioral session, mice were deeply anesthetized with ketamine/xylazine and we performed an electrolytic lesion to aid electrode localization (5 μA current for ∼5s on 2 leads/tetrode), Supertech, IBP-7c). Mice were perfused transcardially, starting with a 2-minute washout period with saline, followed by 4% paraformaldehyde solution for 20 minutes. After the perfusion, mice were removed from the platform and decapitated. The brain was carefully removed and postfixed overnight in 4% PFA. A block containing the full extent of the HDB was prepared and 50 μm thick sections were cut using a Leica 2100S vibratome. All attempts were made to section parallel to the canonical coronal plane to aid track reconstruction efforts. All sections that contained the electrode tracks were mounted on slides in Aquamount mounting medium. Fluorescent and dark field confocal images of the sections were taken with a Nikon C2 confocal microscope. During track reconstruction, it is important to convert the logged screw turns (20 μm for each one eights of a full turn, allowed by a 160 μm pitch custom precision screw, Easterntec, Shanghai) that were performed throughout the experiment into brain atlas coordinates with maximal possible precision. To this end, dark field and bright field images of the brain sections were morphed onto the corresponding atlas planes^87^ using Euclidean transformations only. The aligned atlas images were carried over to fluorescent images of the brain sections showing the DiI-labelled electrode tracks (red) and green fluorescent labeling (cholinergic neurons labelled by the AAV2.5-EF1a-Dio-hChR2(H134R)-eYFP.WPRE.hGh virus) in the target area. The entry points, electrode tips and lesion sites were localized with respect to the atlas coordinates maximizing the combined information of the structural (dark/bright field), DiI track and ChAT-labelling fluoromicrographs. Recording location of each section was interpolated based on the above coordinates, using the screw turn logs and the measured protruding length of the tetrodes after the experiments (also described in^10^). If the track spanned multiple sections, special care was taken to precisely reconstruct the part of the track where the recordings took place within the target area. This procedure minimizes the localization errors that may arise from differences between the recorded and the reference brain coordinates and eliminates the effect of tissue distortions caused by the fixation process. Only those recordings that were convincingly localized to the basal forebrain were analyzed in this study.

### Quantification and statistical analysis

Data processing and analysis was carried out in Matlab R2016a (Mathworks, Natick).

#### Data analysis

Fiber photometry signals were preprocessed according to Lerner et al. (2015). In case of bilateral data acquisition, fiber photometry signal of the side with the better signal-to-noise ratio was chosen for further analysis. Briefly, the fluorescence signals were filtered below 20 Hz using a low-pass Butterworth digital filter to remove high frequency noise. To calculate dff, a least-squares linear fit was applied to the isosbestic 405 nm signal to align its baseline intensity to that of the calcium-dependent 465 nm (f465) signal. The fitted 405 nm signal (f_405,fitted_) was used to normalize the 465 nm signal as follows:

$$dff=\frac{{\text{f}}_{465}-{\text{f}}_{405,fitted}}{{\text{f}}_{405,fitted}}*100$$

to remove the effect of motion and autofluorescence. Slow decay of the baseline activity was filtered out with an 0.2 Hz high pass Butterworth digital filter. Finally, the dff signal was triggered on cue and feedback times, Z-scored by the mean and standard deviation of a baseline window (1s before cue onset) and averaged across trials.

Tetrode recording channels were digitally referenced to a common average reference, filtered between 700-7000 Hz with Butterworth zero-phase filter and spikes were detected using a 750 μs censoring period. Spike sorting was carried out in MClust 3.5 software (A .D. Redish). Autocorrelations were inspected for refractory period violations and putative units with insufficient refractory period were not included in the data set. Cluster separation was measured using Isolation Distance and L-ratio calculated on the basis of two features, the full spike amplitude and the first principle component of the waveform.^88^ Putative single neurons exceeding Isolation Distance (ID) of 20 and below L-ratio of 0.15 were automatically included (n = 20 cholinergic neurons and n = 452 untagged neurons recorded in the same sessions). Additionally, spike sorting was aided by the information provided by light-evoked spike shapes^81^ in n = 5 cholinergic neurons, resulting in a data set of n = 25 optogenetically identified cholinergic neurons (L-ratio of cholinergic neurons, 0.0511 ± 0.0133, median ± SE; ID of cholinergic neurons, 30.0809 ± 6.4121, median ±SE; n = 17, 5, 2, 1 cells in the four mice). Spike shape correlations between spontaneous and light-induced spikes were calculated for all cholinergic neurons. Correlation coefficient exceeded R = 0.85 in all and R = 0.9 in 22/25 optotagged neurons (0.98 ± 0.01, median ±SE; range, 0.87 - 1.0), confirming cholinergic identity.^72^

We did not find any systematic differences in our analyses based on anatomical location; thus, we analyzed the 25 neurons as one dataset. First, we calculated event-aligned raster plots and peri-event time histograms (PETHs) for all neurons. To calculate average PETHs, neuronal responses were triggered on cue and feedback times, Z-scored by the mean and standard deviation of a baseline window (1s before cue onset) and averaged across trials. Response latency and jitter to optogenetic stimulation and behaviorally relevant events were determined based on activation peaks in the peri-event time histograms.^10^ Behavioral performance was tested by comparing the anticipatory lick rate after reward and punishment predicting stimuli in a 1.2 s time window after stimulus onset. Reaction time was determined as the latency of the first lick after stimulus presentation.

We would like to note that an initial analysis of a part of this data set was presented in a bioRxiv preprint (https://www.biorxiv.org/content/10.1101/2020.02.17.953141v1).

#### Model fitting

Firing rates of cholinergic neurons were calculated in 500 ms response windows after cue presentation and 200 ms response windows after reinforcement presentation, to include the full firing response based on the observed time course of cholinergic activation (Figure 4). Firing rates were fitted by the following modified temporal difference RL model of cholinergic activity *(C)*.

$$C=S\cdot \left|R-\eta_{1}E\left(R\right)+P-\eta_{2}E\left(P\right)\right|$$

In this equation, $R-\eta_{1}E\left(R\right)$ stands for reward prediction error (RPE). RPE classically takes the formula of R−E(R), where *E(R)* is expected, and *R* is actual amount of reward at a given time point. This was modified by the *η*_*1*_ parameter, allowing potential differences in sensitivity to reward expectation across animals, sessions and neurons. Similarly, $P-\eta_{2}E\left(P\right)$ represents the difference of expected and encountered punishment, referred to as ‘punishment prediction error’ hereafter. The two terms sum up to a full outcome prediction error, rendered ‘unsigned’ by the absolute value operator. The scaling factor *S* accounts for differences in baseline firing rate of cholinergic neurons. Of note, we found a variable mean firing rate of cholinergic neurons with an average of 8.22 ± 11.39 (SD) Hz during Pavlovian conditioning. We allowed the model to account for this difference with a scalar factor. This implicitly assumes response magnitudes proportional to ‘baseline’ firing rate, as in a multiplicative gain model, similar to what was found for dopamine neurons.^26^ The temporal discounting factor inherent to TDRL models was omitted from the equation, as it leads to only negligible firing rate differences within the few seconds of time that spans a behavioral trial. We note that another way of incorporating differential responsiveness to reward and punishment expectations would be by adding classical learning rates in the form of

$$C=S\cdot \alpha_{1}\left|R-E\left(R\right)\right|+\alpha_{2}\left|P-E\left(P\right)\right|$$

We fitted this alternative model as well; however, this model failed to capture the relative ratios of cue and outcome responses of cholinergic neurons and thus resulted in worse fits than the model presented in Figure 5.

The model was evaluated for the time of the cue, reward and punishment presentations. At the time of cues, *R =* *P* *= 0*, therefore the model takes the form of

$$C=S\cdot \left[\eta_{1}E\left(R\right)+\eta_{2}E\left(P\right)\right]$$

Since no omission responses were observed in cholinergic recordings (Figure S7), we dropped the negative expectation term of the omitted reinforcer at the time of reinforcement (e.g. omitted reward response at the time of punishment), leading to

$$C=S\cdot \left[R-\eta_{1}E\left(R\right)\right]$$

at reward (*P* *= 0*) and

$$C=S\cdot \left[P-\eta_{2}E\left(P\right)\right]$$

at punishment (*R = 0*) delivery. Nevertheless, keeping the omission responses in the model resulted in fits that were statistically indifferentiable (p = 0.86, Wilcoxon signed-rank test of model errors), suggesting that our data were not sufficient to differentiate between RL models with or without omission responses. The *E(R)* and *E(P)* expectation terms were set according to the task contingencies (*E(R)* = 0.8 or 0.25 and *E(P)* = 0.1 or 0.65 for the likely reward and unlikely reward cues, respectively). As a control model, we ran the same fitting process after these contingencies for reward and punishment expectations were swapped (*E(R)* = 0.25 or 0.8 and *E(P)* = 0.65 or 0.1 for the likely reward and unlikely reward cues, respectively). Fitting error was estimated by the maximum likelihood method and minimized by using the fminsearch built-in Matlab function employing the Nelder-Mead simplex algorithm. Models were statistically compared by Wilcoxon signed-rank test on the maximum likelihoods. Note that the compared models had equal complexity and number of parameters; therefore, a punishment term for free parameters was not required. Correlation between model parameters and anticipatory lick rate difference was calculated using the built-in robust regression algorithm of Matlab. Confidence intervals were derived using the polypredci.m function (Star Strider, https://www.mathworks.com/matlabcentral/fileexchange/57630-polypredci, MATLAB Central File Exchange, retrieved December 30, 2020).

Spike trains were simulated as Poisson-processes matched to each recorded cholinergic neuron in frequency (n = 25). Cholinergic responses to cue, reward and punishment were simulated as additional spikes drawn from a Gaussian distribution with fixed latency after the events. The number of ‘evoked spikes’ was based on the best-fit RL model corresponding to each neuron. Peri-event time histograms were generated from simulated spike trains the same way as applied for real data.

#### Statistics

We estimated the sample size before conducting the study based on previous publications, mostly Hangya et al. (2015),^10^ as reported in the Results. Firing rates and other variables were compared across conditions using non-parametric tests, as normality of the underlying distributions could not be determined. Two-sided Wilcoxon signed-rank test was applied for paired, and two-sided Mann-Whitney U-test was applied for non-paired samples. Correlations were estimated by the Pearson’s correlation coefficient, and their significance were judged by using a standard linear regression approach (one-sided F-test, in accordance with the asymmetric null hypothesis of linear regression). The relationship between BFCN firing rate and reaction time quartiles was also assessed by one-way ANOVA. Model fits were compared by negative log likelihood. Since the models compared had equal number of parameters, this is mathematically equivalent with model selection approaches using information criteria (e.g. Akaike and Bayesian Information Criterion). Peri-event time histograms show mean ± SE. Box-whisker plots show median, interquartile range and non-outlier range, with all data points overlaid.

## Data Availability

• Electrophysiology and fiber photometry data have been deposited to a Dryad repository at https://doi.org/10.5061/dryad.p5hqbzkrv. • MATLAB codes generated for this study are available at https://github.com/hangyabalazs/cholinergic_Pavlovian_analysis. • Any additional information required to reanalyze the data reported in this paper is available from the lead contact upon request.
