# Supplementary material for: Cholinergic activity reflects reward expectations and predicts behavioral responses
Source: iScience. 2022 Dec 16;26(1):105814. doi: 10.1016/j.isci.2022.105814 (PMC9830220; doi:10.1016/j.isci.2022.105814)
Supplement: Document S1. Figures S1–S9 [file mmc1.pdf]

iScience, Volume *26*

Supplemental information

Cholinergic activity reflects reward expectations and predicts behavioral responses

Panna Hegedüs, Katalin Sviatkó, Bálint Király, Sergio Martínez-Bellver, and Balázs Hangya

Supplemental figures

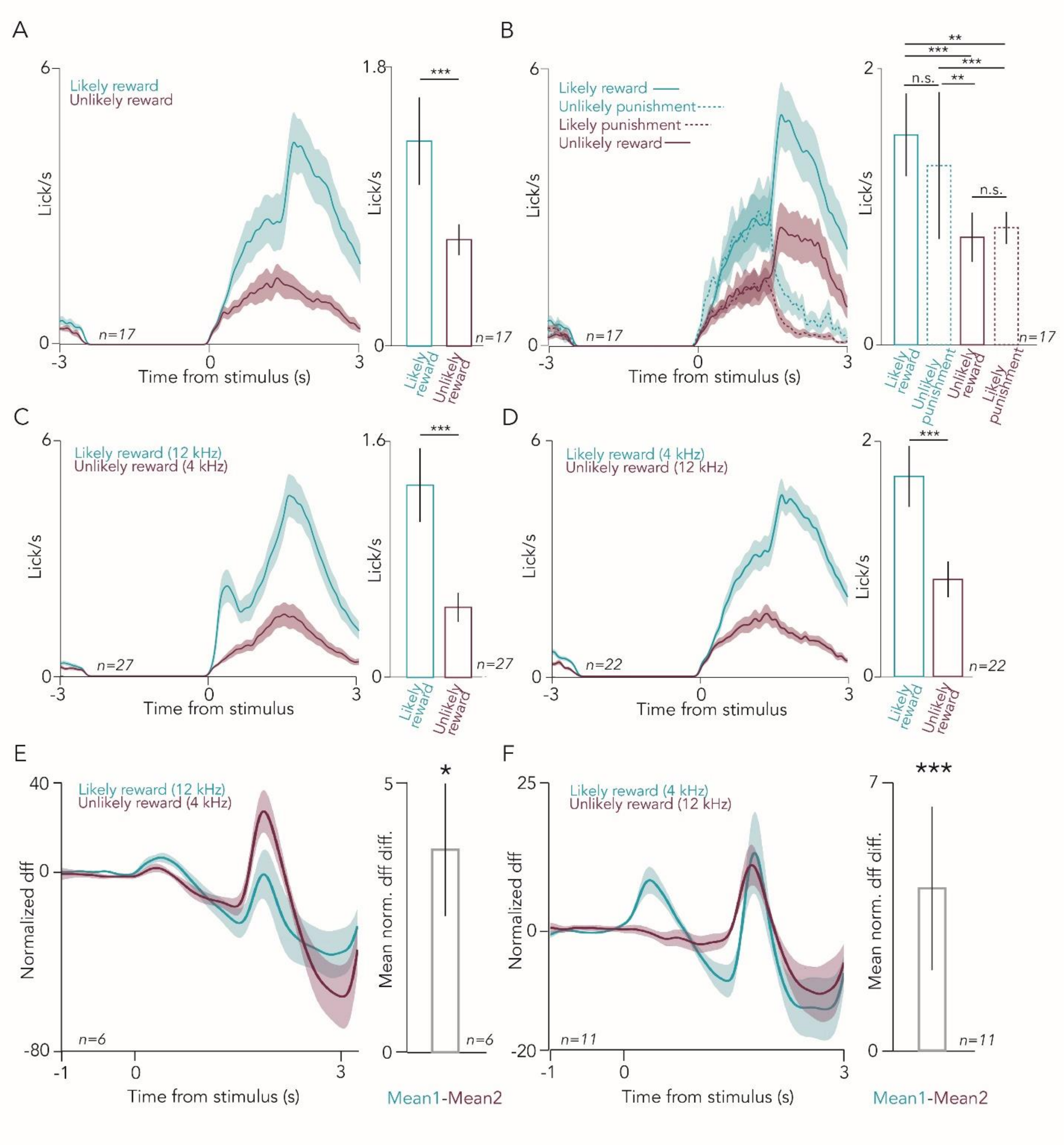

Figure S1. Behavioral performance during bulk calcium imaging of cholinergic neurons, Related to Figure 2. (A) Left, average PETHs of lick responses of all sessions during fiber photometry imaging (n = 17 sessions). Right, statistical comparison of anticipatory lick rates in the RW in likely reward and unlikely reward trials (n = 17 sessions, median ± SE of median, ***, p < 0.001, p = 0.00042, Wilcoxon signed-rank test). (B) Left, average PETHs of lick responses of all sessions of all animals (n = 17 sessions), partitioned

based on the four possible outcomes: expected or surprising reward, expected or surprising punishment. Right, statistical comparison of anticipatory lick rates in the RW with respect to the four possible outcomes (n = 17 sessions, median ± SE of median, **, $p < 0.01$, ***, $p < 0.001$, n.s., $p > 0.05$; from top to bottom, p = 0.00118, p = 0.00085, p = 0.00042, p = 0.7226, p = 0.0012, p = 0.9811, Wilcoxon signed-rank test). (C) Left, average PETHs of lick responses in sessions where likely reward (unlikely punishment) was predicted by a 12 kHz tone and unlikely reward (likely punishment) was predicted by a 4 kHz tone (n = 27 sessions, all sessions with full task contingencies were included). Right, statistical comparison of anticipatory lick rates in the RW in likely reward and unlikely reward trials (n = 27 sessions, median ± SE of median, ***, $p < 0.001$, $p = 2.91 \times 10^{-5}$, Wilcoxon signed-rank test). (D) The same as C, but with opposite contingencies (n = 22 sessions, all sessions with full task contingencies were included; median ± SE of median, ***, $p < 0.001$, p = 0.00033, Wilcoxon signed-rank test). (E) Left, average PETH of Z-scored dff aligned to outcome-predicting conditioned stimuli, where likely reward (unlikely punishment) was predicted by a 12 kHz tone and unlikely reward (likely punishment) was predicted by a 4 kHz tone (n = 6 sessions). Right, bar graph of average normalized difference in dff after likely reward and unlikely reward cues. Median ± SE of median, *, $p < 0.05$, p = 0.03125, Wilcoxon signed-rank test. (F) The same as E, but with opposite contingencies (n = 11 sessions, median ± SE of median, ***, $p < 0.001$, p = 0.00098, Wilcoxon signed-rank test).

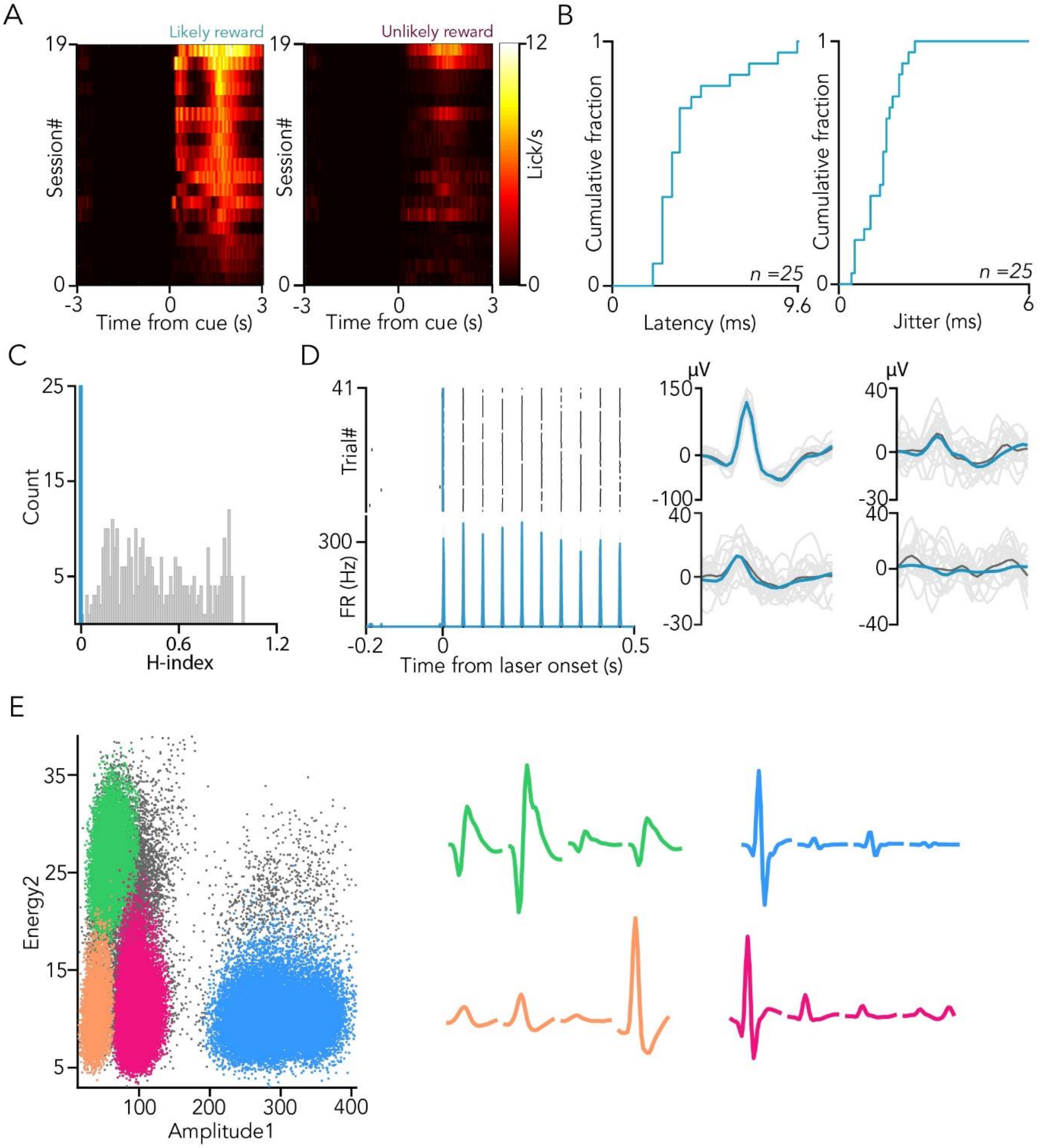

Figure S2. Optogenetic tagging of cholinergic neurons, Related to Figure 3. (A) Color-coded PETHs of lick responses of all sessions in which cholinergic neurons were recorded (n = 19 sessions; black, no licks; white, maximal lick response). (B) Left, cumulative histogram of the peak response latency of BFCNS after optogenetic stimulation. Right, cumulative histogram of the jitter of cholinergic spike responses after optogenetic stimulation. (C) Distribution of the significance values of the SALT statistical test (H-index) for

all recorded neurons (blue, $p < 0.01$, tagged cholinergic neurons; grey, $p > 0.01$, untagged neurons). (D) Left, example spike raster (top) and PETH (bottom) of an optogenetically tagged BFCN responding to 20 Hz blue laser light stimulation. Right, average spike waveform of the same BFCN on the four tetrode channels (blue, average light-evoked spikes; black, average spontaneous spikes; grey, all spikes). (E) Left, example of spike clusters plotted in feature space from a recording session. Right, average spike waveform of the recorded neurons on each tetrode channel.

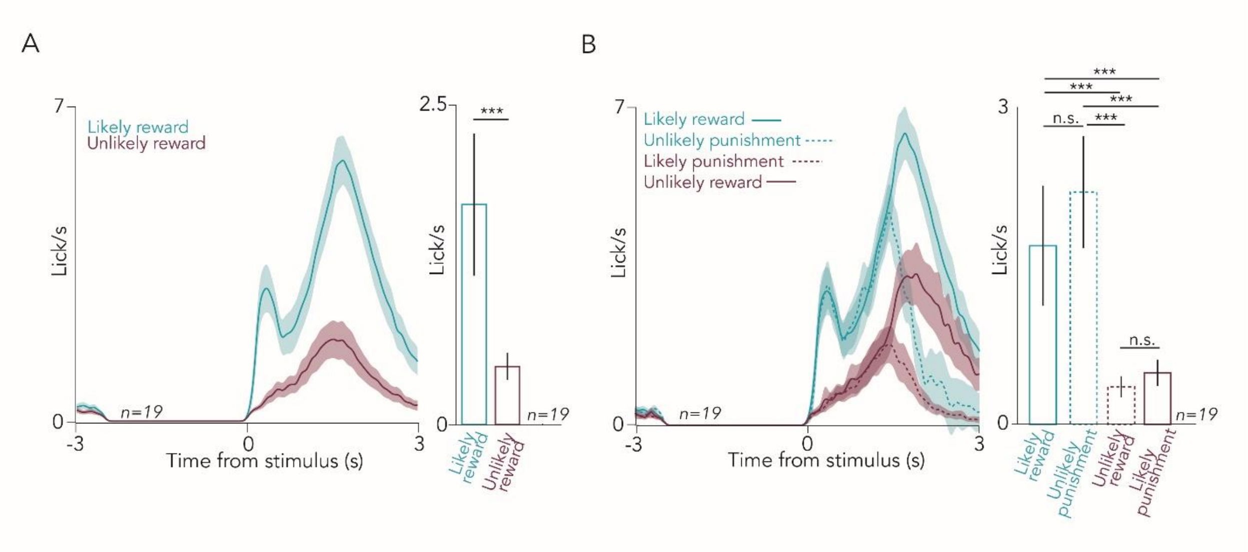

Figure S3. Behavioral performance during tetrode recordings, Related to Figure 3. (A) Left, average PETHs of lick responses of all sessions of all animals (n = 19 sessions). Right, statistical comparison of anticipatory lick rates in the response window (RW) in likely reward and unlikely reward trials (median ± SE of median, n = 19 sessions; ***, $p < 0.001$, $p = 0.00034$, Wilcoxon signed-rank test). (B) Left, average PETHs of lick responses of all sessions of all animals (n = 19 sessions), partitioned based on the four possible outcomes: expected or surprising reward, expected or surprising punishment. Right, statistical comparison of anticipatory lick rates in the RW, with respect to the four possible outcomes (median ± SE of median, n = 19 sessions; ***, $p < 0.001$, n.s., $p > 0.05$; from top to bottom, $p = 0.00034$, $p = 0.00084$, $p = 0.00021$, $p = 0.8721$, $p = 0.00025$ and $p = 0.9512$, Wilcoxon signed-rank test).

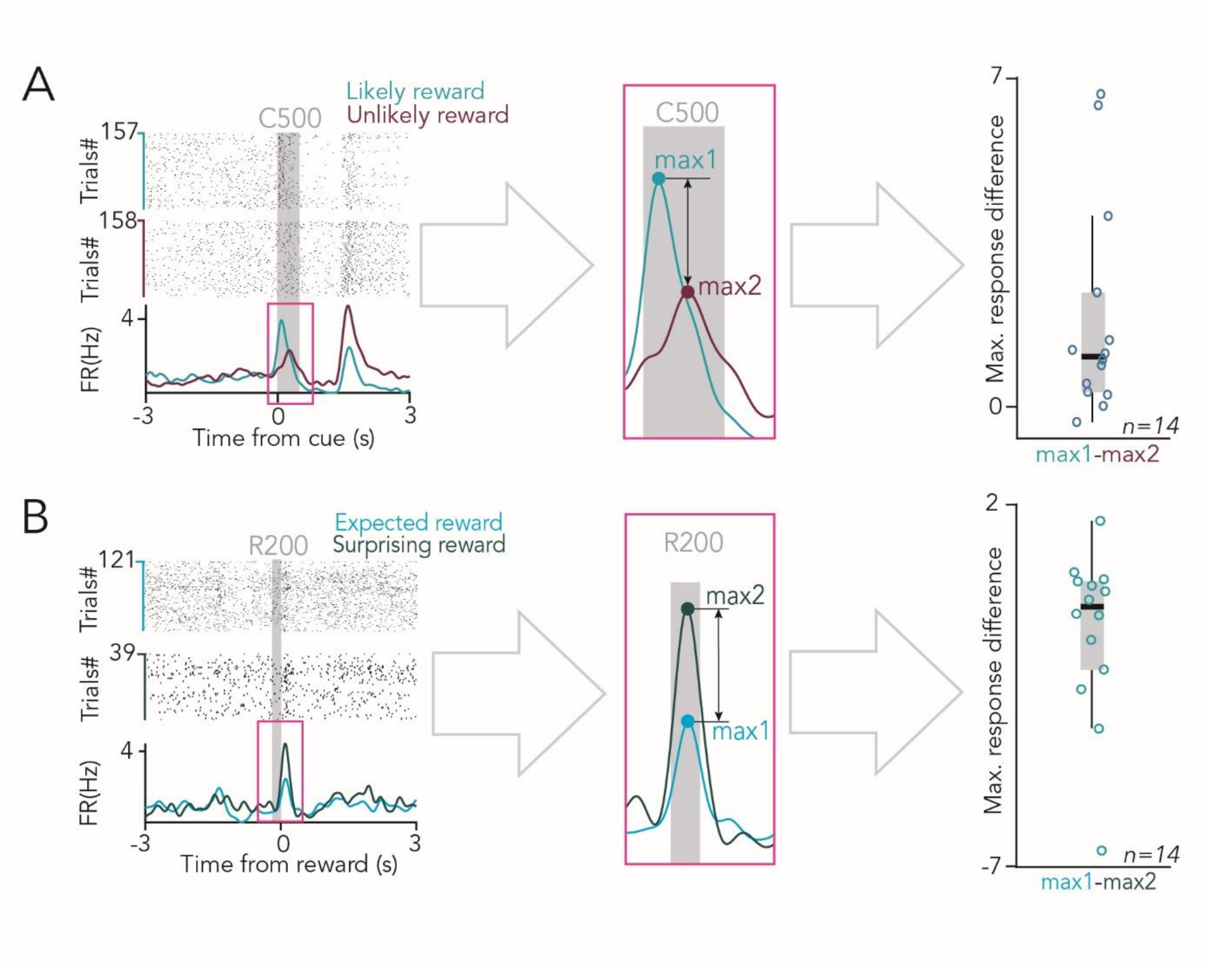

Fig S4. Schematic illustration of quantifying neuronal responses, Related to Figure 4. (A) Schematic illustration of quantifying neuronal response to conditioned stimuli. Peak firing rate of the PETH and total spike number were calculated in a 500 ms window after cue presentation (C500), based on the range of response latencies (see Results). (B) Schematic illustration of quantifying neuronal responses to reward. Peak firing rate of the PETH and total spike number were calculated in a 200 ms window after reward delivery (R200), based on the range of response latencies (see Results). Both maximal response and average firing rate (FR) were statistically compared using Wilcoxon signed-rank test. Box-whisker plots show median, inter-quartile range and non-outlier range.

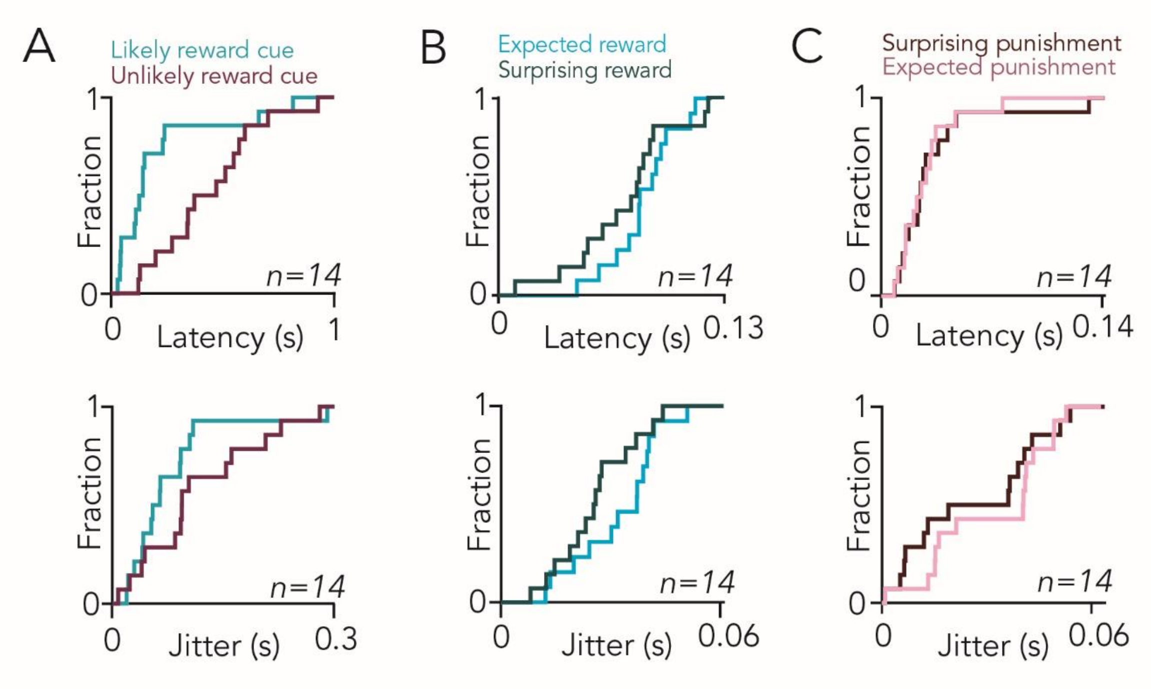

Fig S5. Latency and jitter of identified cholinergic neurons, Related to Figure 4. (A) Top, Cumulative histogram of the peak response latencies of BFCNs (n = 14) after cue presentation. Bottom, Cumulative histogram of the jitter of cholinergic spike responses after cue presentation (n = 14 BFCNs). (B) Top, Cumulative histogram of the peak response latencies of BFCNs (n = 14) after reward. Bottom, Cumulative histogram of the jitter of cholinergic spike responses after reward (n = 14 BFCNs). (C) Top, Cumulative histogram of the peak response latencies of BFCNs (n = 14) after punishment. Bottom, Cumulative histogram of the jitter of cholinergic spike responses after punishment (n = 14 BFCNs).

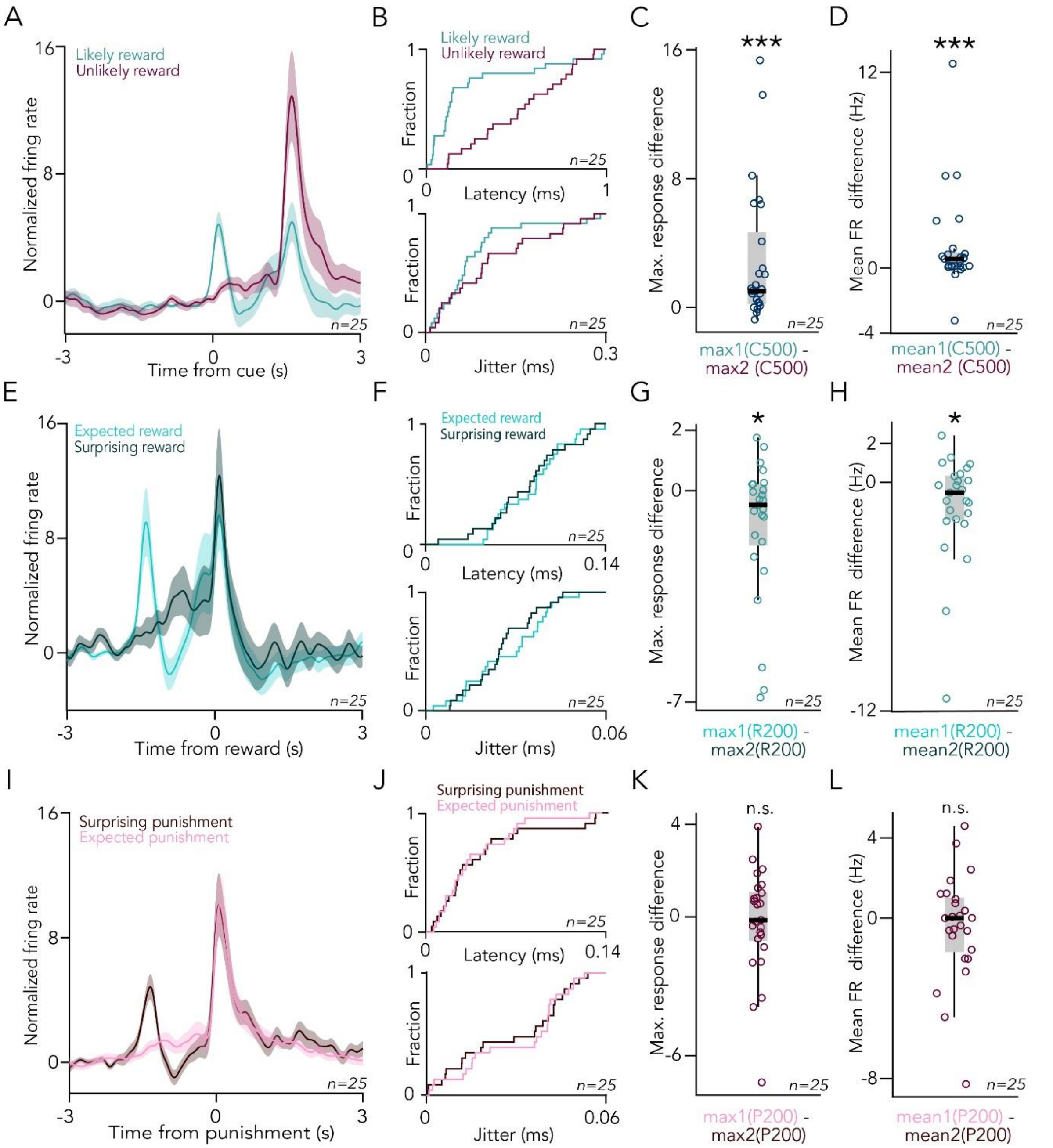

Figure S6. Cholinergic neurons respond more to reward-predicting cues and surprising reward, Related to Figure 4. (A) Average cue-aligned PETHs of identified BFCNs (errorshade, mean ± SE; n = 25), aligned to cue onset, separately for the cues predicting likely reward (turquoise) vs. unlikely reward (purple). (B) Top, Cumulative histogram of the peak response latencies of BFCNs (n = 25) after cue presentation. Bottom, Cumulative histogram of the jitter of cholinergic spike responses after cue presentation (n = 25 BFCNs).

(C) Difference in peak response after cues predicting likely reward and those predicting unlikely reward. ***, $p < 0.001$, $p = 0.0001743$, Wilcoxon signed-rank test, $n = 25$. (D) Difference in mean firing rate after cues predicting likely reward and those predicting unlikely reward. ***, $p < 0.001$, $p = 0.000664$, Wilcoxon signed-rank test, $n = 25$. (E) Average reward-aligned PETHs of identified BFCNs (errorshade, mean ± SE; $n = 25$), separately for rewards after the cue predicting likely reward (light turquoise, expected reward) vs. after the cue predicting unlikely reward (dark turquoise, surprising reward). (F) Top, Cumulative histogram of the peak response latencies of BFCNs ($n = 25$) after reward delivery. Bottom, Cumulative histogram of the jitter of cholinergic spike responses after reward delivery ($n = 25$ BFCNs). (G) Difference in peak response after expected and surprising rewards. *, $p < 0.05$, $p = 0.02465$, Wilcoxon signed-rank test, $n = 25$. (H) Difference in mean firing rate after expected and surprising rewards. *, $p < 0.05$, $p = 0.03704$, Wilcoxon signed-rank test, $n = 25$. (I) Average punishment-aligned PETHs of identified BFCNs (errorshade, mean ± SE; $n = 25$), separately for punishments after the cue predicting likely reward (dark purple, surprising punishment) vs. after the cue predicting unlikely reward (light purple, expected punishment). (J) Top, Cumulative histogram of the peak response latencies of BFCNs ($n = 25$) after punishment delivery. Bottom, Cumulative histogram of the jitter of cholinergic spike responses after punishment delivery ($n = 25$ BFCNs). (K) Difference in peak response after expected and surprising punishments. n.s., $p > 0.05$, $p = 0.94637$, Wilcoxon signed-rank test, $n = 25$. (L) Difference in mean firing rate after expected and surprising punishments. n.s., $p > 0.05$, $p = 0.54851$, Wilcoxon signed-rank test, $n = 25$. All box-whisker plots in the figure show median, inter-quartile range and non-outlier range.

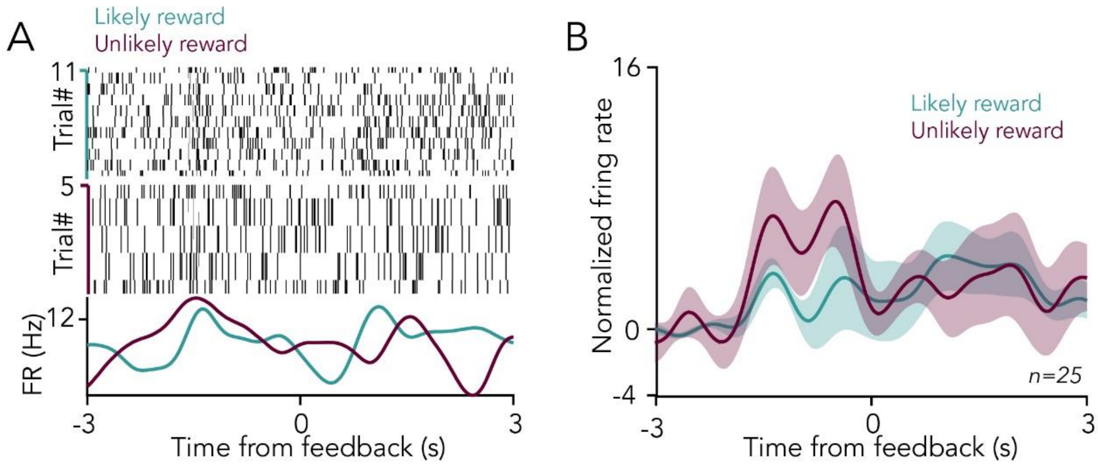

Figure S7. Cholinergic neurons do not respond to outcome omissions, Related to Figure 4. (A) Example spike raster and PETH of a BFCN aligned to the expected time of omitted feedback. **(B)** Average PETH of all BFCNS (errorshade, mean ± SE; n = 25) with the same alignment.

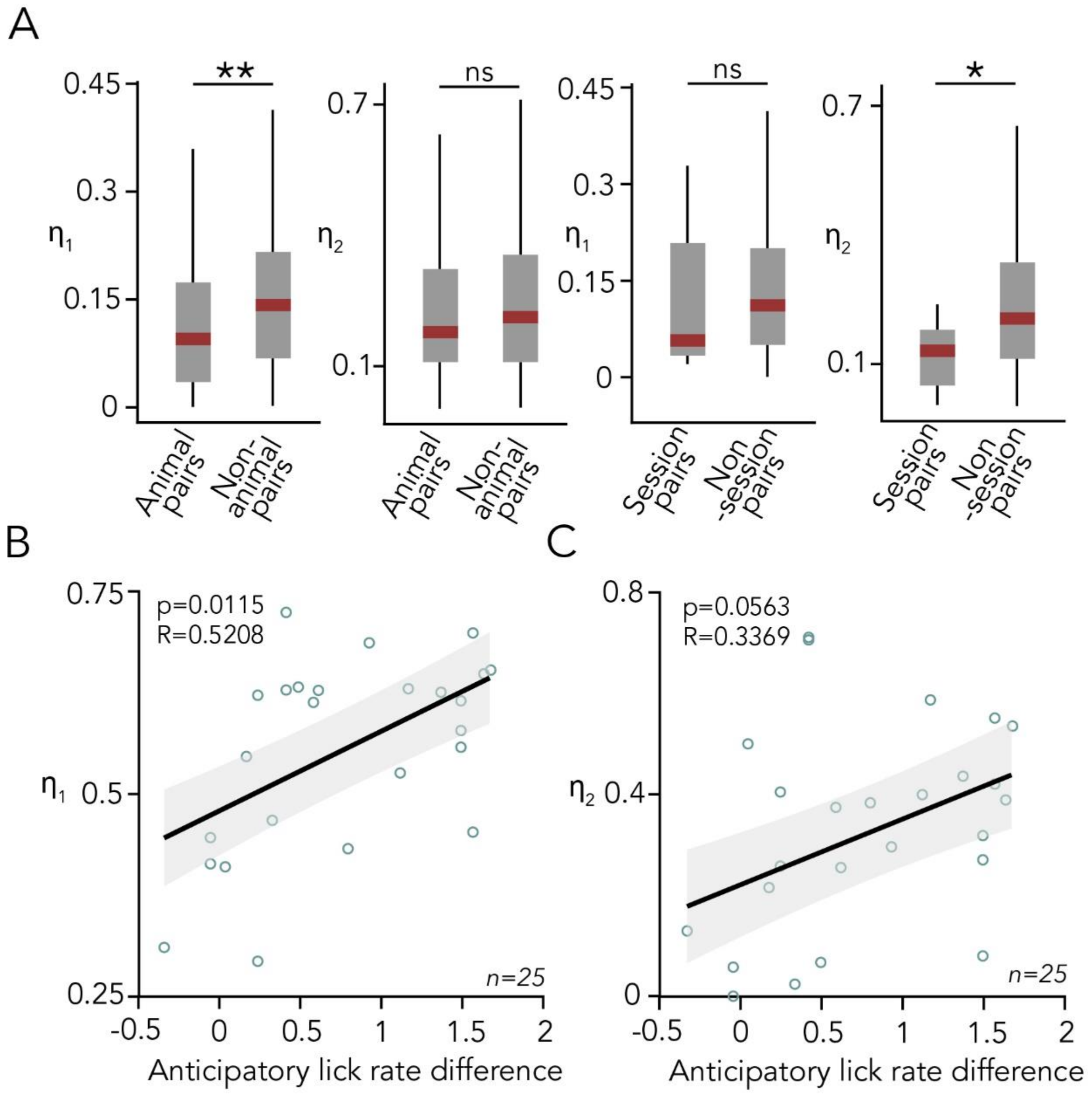

Figure S8. Prediction error parameters reveal behavioral correlations, Related to figure 5. (A) Left, difference of best-fit η_1 and η_2 parameters between BFCNs recorded in the same animal vs. in different mice. Right, difference of best-fit η_1 and η_2 parameters between BFCNs recorded in the same recording session vs. in different sessions. *, $p < 0.05$; **, $p < 0.01$, n.s., $p > 0.05$, Mann-Whitney U-test, n = 147 animal pairs, n = 153 non-animal pairs, n = 7 session pairs, n = 293 non-session pairs; exact p values, p = 0.00199, p = 0.2496, p = 0.5725, p = 0.04677, respectively. Box-whisker plots show median, inter-quartile range and non-outlier range. **(B)** Correlation between the best-fit η_1 parameter and the difference in anticipatory lick rate after likely reward vs. unlikely reward cues (R = 0.5208, Pearson's correlation coefficient; p = 0.0115, linear regression, F-test). Correlation between the best-fit η_2 parameter and the

difference in anticipatory lick rate after likely reward vs. unlikely reward cues (R = 0.3369, Pearson's correlation coefficient; p = 0.0563, linear regression, F-test).

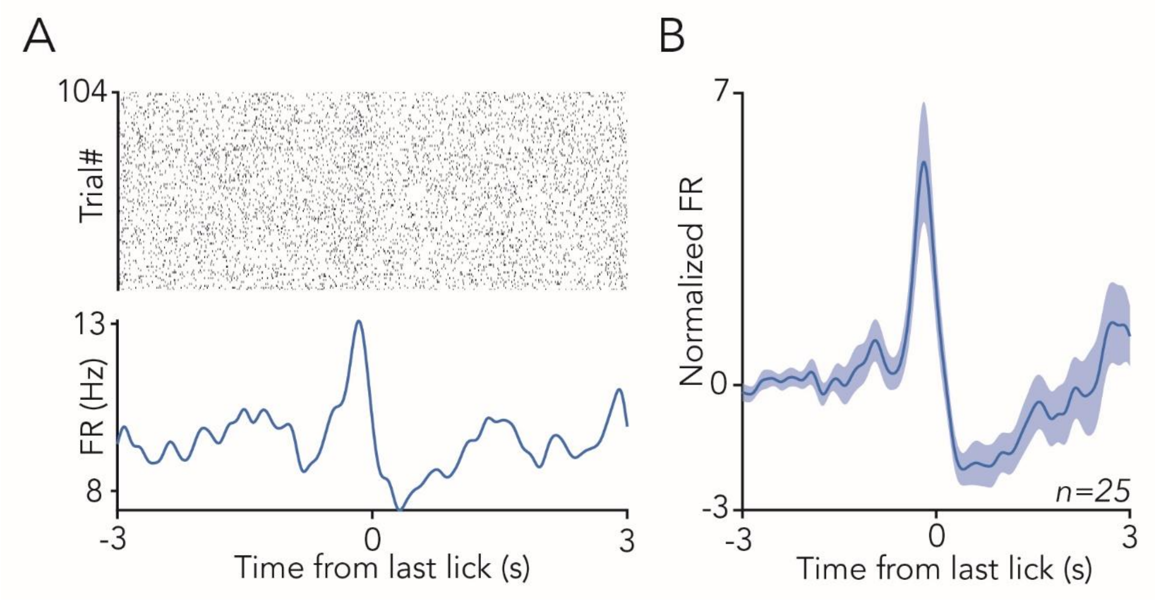

Figure S9. Cholinergic activity aligned to the last lick before the foreperiod, Related to Figure 6. (A) Raster plot (top) and PETHs (bottom) of an example cholinergic neuron aligned to the last lick before the foreperiod. (B) Average PETH of all cholinergic neurons (errorshade, mean ± SE, n = 25) aligned to the last lick before the foreperiod. FR, firing rate.
